# Trio analysis in dystonia identifies *de novo KLC1* variants in a kinesinopathy with distinct motor and neurodevelopmental features

**DOI:** 10.1016/j.ebiom.2026.106358

**Published:** 2026-06-29

**Authors:** Elisa Peirano, Laura O’Regan, Philip Harrer, Ivana Dzinovic, Magda S. Chegkazi, Petra Havrankova, Theresa Brunet, Rossella Capolino, Claudia Cesario, Stefania Ferro, Eva Hammar, Russia Hà-Vinh Leuchter, Elisabetta Indelicato, Maureen Jacob, Lukas Kunc, Henri Margot, Oriano Marin, Maria Mazurkiewicz-Bełdzińska, Niccolò E. Mencacci, Antonio Novelli, Laura Orec, Michael Poschmann, Alexandra Sitzberger, Ugo Sorrentino, Melanie Spanjaard, Matias Wagner, Magdalena Krygier, Sylvia Boesch, Jan Necpal, Matej Skorvanek, Donald L. Gilbert, Robert Jech, Mark Dodding, Roberto A. Steiner, Michael Zech

**Affiliations:** a Department of Biomedical Sciences, University of Padova, Padova, Italy; b School of Biochemistry and Biomedical Sciences, Faculty of Health and Life Sciences, University of Bristol, Bristol, BS8 1TD, UK; c School of Medicine and Health, Institute of Human Genetics, Technical University of Munich, Munich, Germany; d Institute of Neurogenomics, Helmholtz Zentrum München, Munich, Germany; e Randall Centre of Cell and Molecular Biophysics, Faculty of Life Sciences and Medicine, King’s College London, London, UK; f Department of Neurology, 1st Faculty of Medicine and General University Hospital in Prague, Charles University, Prague, Czech Republic; g Medical Genetics Unit, Bambino Gesù Children’s Hospital, IRCSS, Rome, Italy; h Laboratory of Medical Genetics, Translational Cytogenomics Research Unit, Bambino Gesù Children’s Hospital, IRCCS, Rome, Italy; i Division of Genetic Medicine, Geneva University Hospitals, Geneva, 1205, Switzerland; j Division of Development and Growth, Department of Paediatrics, Gynaecology and Obstetrics, Geneva University Hospitals and University of Geneva, Switzerland; k Centre for Rare Movement Disorders Innsbruck, Department of Neurology, Medical University of Innsbruck, Innsbruck, Austria; l Genetic Medicine Service, Department of Diagnostics, Centre for Medical Genomics, Geneva University Hospitals, Geneva, Switzerland; m Department of Developmental Neurology, Medical University of Gdansk, Gdansk, Poland; n Department of Neurology, Northwestern University Feinberg School of Medicine, Chicago, IL, USA; o Division of Paediatric Neurology and Metabolic Medicine, Centre for Child and Adolescent Medicine, University Hospital Heidelberg, Heidelberg, Germany; p Schön Klinik München Harlaching, Munich, Germany; q Division of Paediatric Neurology and Developmental Medicine and LMU Centre for Children with Medical Complexity, Dr. von Hauner Children’s Hospital, LMU Hospital, Ludwig-Maximilians-Universität, Munich, Germany; r Institute of Human Genetics, Heidelberg University, Heidelberg, Germany; s Department of Neurology, Zvolen Hospital, Zvolen, Slovakia; t Parkinsonism and Movement Disorders Treatment Center, Zvolen Hospital, Zvolen, Slovakia; u Department of Neurology, P.J. Safarik University, Kosice, Slovakia; v Department of Clinical Neurosciences, P.J. Safarik University, Kosice, Slovakia; w Department of Neurology, University Hospital of L. Pasteur, Kosice, Slovakia; x Division of Neurology, Cincinnati Children’s Hospital Medical Center, Cincinnati, OH, 45229, USA; y Institute for Advanced Study, Technical University of Munich, Garching, Germany; z Department of Pediatrics, University of Cincinnati College of Medicine, Cincinnati, OH, 45221, USA

**Keywords:** *De novo* variants, Kinesins, KLC1, Movement disorder, Dystonia, Neurodevelopmental disorder

## Abstract

**Background:**

Although *de novo* causation in dystonia is widely acknowledged, there have been only a few trio-sequencing analyses in this field. We sought to prioritise *de novo* variants in dystonia and characterise the clinical and molecular features associated with the top gene candidate identified after genomic matchmaking.

**Methods:**

We (re)assessed exome-sequencing data for *de novo* variants in genes with strong mutational constraint in a sample of 257 dystonia trios. Via data sharing, we collected information on individuals with variants in *KLC1*, encoding a subunit of the axonal-transport motor protein kinesin-1. Biophysical, biochemical, and functional studies, including differential scanning fluorimetry, X-ray crystallography, fluorescence-polarisation measurements, and immunoprecipitation from cells were performed for representative *KLC1* variants.

**Findings:**

Missense and loss-of-function *de novo* variants in constrained genes without implication in autosomal dominant or X-linked conditions were found in 11.7% (30/257) of cases with dystonia. We then ascertained 7 unrelated patients with movement and neurodevelopmental disorders who harboured distinct, predicted deleterious *de novo KLC1* missense variants. These variants clustered within the cargo adaptor-binding tetratricopeptide repeat domain and 3 variants mapped to an identical amino-acid position. Highly similar infantile-onset dystonic-spastic phenotypes were observed in the subjects with the recurrently affected residue. For all functionally tested variants, we observed changes in KLC1 stability and/or altered binding behaviour to known kinesin-1 interactors, such as JIP3, previously associated with dystonia and neurodevelopmental impairment.

**Interpretation:**

Our research supports the existence of a kinesinopathy linked to *KLC1*, featuring phenotypic overlap with diseases related to mutational defects of key interactors of KLC1. The full dystonia *de-novo* variant compendium is reported as a resource for additional disease-gene discovery.

**Funding:**

Else Kröner-Fresenius-Stiftung, German Federal Ministry of Education and Research, Technical University of Munich–Institute for Advanced Study, EU Renewal and Resilience Plan, Czech Ministry of Health, European Union—Next Generation EU, Italian Ministry for Universities and Research.

**Research in context**

**Evidence before this study**

While *de novo* mutations are recognised as a significant cause of dystonia, systematic trio-sequencing studies in this field remain scarce. Prior work has identified individual genetic causes, but large-scale prioritisation and reporting of *de novo* variants in unexplained cases is limited. Specifically, the role of *KLC1*—encoding a subunit of the kinesin-1 motor protein essential for axonal transport—had not yet been established as a contributor to autosomal dominant movement and neurodevelopmental disorders, although mutations in its interaction partner JIP3 (MAPK8IP3) were known to cause similar phenotypes.

**Added value of this study**

Through exome-sequencing of 257 dystonia trios and international data sharing, we identified *KLC1* as a disease-associated gene. We report seven unrelated individuals harbouring deleterious *de novo* missense variants that cluster within the cargo-binding TPR domain of KLC1. Our study characterises a distinct infantile-onset dystonic-spastic phenotype, particularly linked to a recurrently affected amino-acid residue. Using comprehensive biophysical and functional studies including X-ray crystallography and different complementary biochemical assays, we demonstrate that these variants destabilise the KLC1 protein or disrupt its ability to bind key cargo adaptors like JIP1 and JIP3. Additionally, we provide a comprehensive compendium of *de novo* variants from our cohort as a resource for additional gene discovery.

**Implications of all the available evidence**

This research provides strong evidence for a previously unrecognised kinesinopathy associated with *de novo KLC1* missense variants, expanding the molecular spectrum of transport-related neurodevelopmental movement disorders. The resemblance in clinical presentation between KLC1 defects and mutational impairment of its interactors (like JIP3) underscores how vital the kinesin-1 machinery is for motor function and opens new avenues for therapeutic intervention. Our findings suggest that *KLC1* should be included in the diagnostic workup for early-onset dystonia and spasticity, particularly when *de novo* causation is suspected.

## Introduction

*De novo* variation in genes classified as intolerant to missense and loss-of-function variants has a major contribution to neurogenetic disease.^1^^,^^2^ Exome and genome sequencing strategies were successfully employed in analyses of *de novo* variants in large cohorts of individuals affected by developmental delay, intellectual disability, and epilepsy.^1^, ^2^, ^3^ Frameworks for variant interpretation, including the American College of Medical Genetics and Genomics (ACMG) classification guidelines and ClinGen gene curation criteria, have further defined the evidence thresholds required to establish robust gene–disease associations.^4^^,^^5^ In contrast to the broader categories of developmental disorders, we understand far less about the implication of *de novo* variants in phenotypes for which the results from sequenced trios have not yet been systematically reported.^6^ The involvement of *de novo* variants in functionally important genes that are depleted or constrained for variation is already established in dystonia,^7^, ^8^, ^9^ but few existing studies have catalogued such genomic changes found in patients with dystonic diseases and a number of associated conditions likely await discovery. The genetic underpinnings of dystonia are highly heterogeneous, as demonstrated by recent large-scale sequencing efforts.^7^, ^8^, ^9^

Several recent investigations have demonstrated that variant-constrained genes encoding members of the kinesin superfamily proteins (KIFs) are frequently mutated in neurodevelopmental and neurodegenerative syndromes.^10^, ^11^, ^12^ Kinesins are evolutionarily conserved molecular motors mediating essential intracellular transport functions.^10^ For example, *de novo* variants in the kinesin-3 family gene *KIF1A* (*Z* score = 5.04; gnomAD-v4.1.0)^13^ cause a variety of neurological presentations ranging from neurodevelopmental impairment to specific movement disorders,^14^ including dystonia^15^ (HSP/DYT-*KIF1A*; OMIM:614255). One of the most extensively characterised KIF family members is kinesin-1, which is formed by hetero-tetramers of 2 kinesin heavy chain subunits (kinesin family member 5s, KIF5s, KIF5A-C) and 2 kinesin light chain subunits (KLCs, KLC1-4).^16^^,^^17^ Kinesin-1 is required for anterograde delivery of neurotransmitter receptors, mitochondria, lysosomes, and other axonal cargos in a microtubule-dependent manner and thus plays crucial roles in brain development and synaptic plasticity.^18^ Although a compositionally diverse array of kinesin-1 complexes are assembled in the brain, expressions of *KLC1* and its heavy-chain binding partner *KIF5A* are enriched in neurons. Together, they support the attachment of diverse vesicular cargos to kinesin-1 via specialised adaptors and other interactors,^17^ such as JIP1, JIP3 (also known as MAPK8IP3), SKIP (PLEKHM2),^19^ and the dystonia-associated protein TorsinA.^20^ While *KIF5A* (*Z* score = 5.04; gnomAD-v4.1.0)^13^ has been linked to spastic paraplegia and other inherited disturbances of the motor system (HSP-*KIF5A;* OMIM:604187),^14^^,^^21^ *KLC1* has remained unassociated with a Mendelian condition despite its pronounced mutational constraint (*Z* score = 3.93; LOEUF = 0.51; gnomAD-v4.1.0).^13^

In this study, we extracted *de novo* variants from whole-exome sequencing (WES) datasets of 257 genetically unresolved dystonia-affected individuals and their parents. We focused on missense and loss-of-function variations affecting genes with significant constraint scores.^13^ We present the molecular and clinical findings for 7 patients with *de novo* missense variants in *KLC1*, identified through the *de novo* variant discovery effort in dystonia and ensuing matching of cases with similar genotypic and clinical profiles. We characterise the shared features of these individuals, including strikingly overlapping neurodevelopmental movement disorders with dystonia and spasticity linked to a recurrently mutated *KLC1* amino-acid residue. Using an interdisciplinary approach encompassing biophysical, biochemical, and functional analyses, we provide compelling evidence for a role of *KLC1* variants as the cause of a previously unrecognised early-onset neurodysfunction syndrome.

## Methods

### *De novo* variant analysis in dystonia and genomic matchmaking

The dystonia-affected child-parent trio series (*n* = 257 trios) (re)investigated in this work was part of a previously described, ongoing genomic research study, recruited internationally at specialty centres for movement and neurodevelopmental disorders between 2015 and 2025.^7^^,^^8^ We included the trio WES datasets of the families from our study who had been already extensively screened and found to be negative for likely pathogenic or pathogenic variants in known disease genes^7^^,^^8^ (Supplementary Fig. 1A and B). This series encompassed index patients with diverse dystonic phenotypes, subsumed under two categories: (1) isolated dystonia, i.e., individuals with dystonia as the sole clinical manifestation (*n* = 58); and (2) combined dystonia, i.e., individuals with presentations which had a prominent component of dystonia but were associated with other neurological and/or systemic abnormalities (*n* = 199).^22^ The processing of WES data and all variant annotations for the trio (re)analyses were performed as detailed earlier.^7^^,^^8^ We identified rare *de novo* variants by excluding those variants present in the WES data of the parents and those observed with a minor allele frequency of ≥0.001 (gnomAD-v4.1.0 and 30,000 internal exomes) by using custom in-house scripts^7^^,^^8^ (Supplementary Fig. 1A). Only variants with positions covered ≥20× and supported by ≥20% of the total reads at the called position were retained. We focused our attention on *de novo* non-synonymous exonic and canonical splice-site variants in variation-constrained genes (canonical transcripts)^13^ without documented associations to autosomal dominant/X-linked disorders,^14^ as follows: (1) missense SNVs in genes with *Z* score ≥3.09 (gnomAD-v4.1.0)^13^; and (2) predicted loss-of-function SNVs and indels in genes with LOEUF <0.6 (gnomAD-v4.1.0).^13^ The thresholds were selected based on the gnomAD v4 constraint framework.^13^ Missense Z scores quantify the deficit of observed missense variants relative to a sequence-context–dependent mutational model, with a Z score ≥3.09 indicating a statistically significant depletion of amino acid-changing variation consistent with purifying selection. The LOEUF score provides a continuous, statistically robust measure of loss-of-function intolerance; a value < 0.6 captures genes under strong evolutionary constraint against loss-of-function variation in the gnomAD v4 dataset.^13^ For initial candidate prioritisation, CADD^23^ scores were also considered for identified *de novo* variants. For *KLC1* variants, additional *in silico* predictions were obtained, including REVEL,^24^ AlphaMissense,^25^ and MetaDome^26^ outputs; *KLC1* variant interpretation followed ACMG guidelines for computational evidence (PP3).^4^ We visually verified all prioritised *de novo* variants using the Integrative Genomics Viewer (IGV). Aiming to find supportive evidence for the candidacy of a gene of interest, we queried collaborative networks,^7^^,^^8^ online matchmaking platforms,^27^ as well as published compendia for *de novo* mutations.^1^^,^^2^^,^^28^ On the basis of these strategies, we gathered a group of 7 patients harbouring *KLC1 de novo* missense variants: individual 1 through the dystonia trio-WES series^7^^,^^8^; individuals 2, 3, and 4 through direct collaboration with colleagues^29^^,^^30^; individuals 5 and 6 through GeneMatcher^27^; and a seventh subject (individual 7 in this study) who was previously reported in the frame of a meta-analysis of *de novo* variants in neurodevelopmental disorders.^1^^,^^2^^,^^28^ Individuals 2–6 were analysed by trio WES or trio whole-genome sequencing in research and diagnostic settings as previously reported (Supplementary Methods),^29^, ^30^, ^31^, ^32^ and all were identified to have a *KLC1* candidate variant after exclusion of underlying known genetic causes for their conditions. To delineate the clinical spectrum associated with *KLC1* variants, we ascertained information by inviting patients back to the clinic and performing in-depth phenotyping including neurologic, developmental, behavioural, and dysmorphology assessment.

### Expression constructs and mutagenesis

For biophysical studies of selected *KLC1* missense variants that we collected for affected individuals, mutant proteins were generated starting from a commercially provided (Tsinkge Biotech, China) codon-optimised DNA sequence for bacterial expression encoding the N-terminally-extended tetratricopeptide repeat (TPR) domain of mouse KLC1 including the conserved leucine-phenylalanine-proline motif (residues 172–496, Uniprot Q5UE59, KLC1^extTPR^) inserted between the NdeI/BamHI sites of a pET28 vector (Novagen, Germany). Mouse and human TPR domains show high sequence homology (Supplementary Fig. 2). Five of the 7 identified *KLC1* variants were selected for functional characterisation: p.(Asp253Ala), p.(Asp253Gly), p.(Met257Val), p.(Ser389Phe), and p.(Leu470Phe); these were chosen to achieve coverage of distinct structural regions of the TPR domain implicated by patient variants, including TPR2 (Asp253, Met257), TPR5 (Ser389), and TPR6 (Leu470). The selected 5 variants therefore provided representative coverage of the affected protein regions. An additional TEV-cleavable His_6_-tag was included to facilitate purification. Mutagenesis was performed using the Q5 site-directed mutagenesis kit (New England Biolabs, USA; RRID:SCR_013517, cat. No. E0554S) with the following set of primers: D253A_f: CGACCATCCAGCCGTTGCGACC, D253A_r: TGACCAGAGGTCTTCTCCAGG, D253G_f: CGACCATCCAGGCGTTGCGACCA, D253G_r: TGACCAGAGGTCTTCTCCAGGTC, M257V_f: CGTTGCGACCGTGCTGAACATTC, M257V_r: TCTGGATGGTCGTGACCAGA, S389F_f: CAACCTCGCTTTTTGCTACCTGA, S389F_r: TTCTTGGTCTTCGCAACG, L470F_f: CTTGAAGAACTTTGGTGCTCTGTAC, L470F_r: GTAGTGGTTACGGTC. All constructs were validated by DNA sequencing. For crystallographic studies, a codon-optimised DNA sequence encoding KLC1^extTPR^ fused to the C-terminal eleven amino acids of human JIP1 (residues 701–711, Uniprot Q9UQF2, JIP1^C-term^) linked via a (Thr-Gly-Ser)_10_ flexible connector was purchased from Genscript Biotech (Oxford, UK; RRID:SCR_002891) and subcloned between the NdeI/XhoI sites of a pET28 vector (Novagen). This strategy allowed for the expression of a chimeric protein that was identified as KLC1^extTPR^-JIP1^C-term^, bearing a thrombin-cleavable N-terminal His_6_ tag. A high-affinity nanobody for KLC1^extTPR^, described in a previous publication,^17^ was generated by the VIB Nanobody Service Facility of Vrije Universiteit Brussel (Brussels, Belgium; RRID:SCR_011769) following the immunisation of a llama. The antigen-specific nanobody supplied in a pMECS vector was sub-cloned between the PstI/BstEII sites of a pHEN6c vector. This allowed the expression of C-terminally His_6_-tagged nanobody in the periplasmic space of *E. coli*. pCB6-HA-KLC1^TPR^ (amino acids 205–496 from mouse) used for immunoprecipitation was described previously.^17^ Site-directed mutagenesis was performed to introduce selected patient-derived missense variants, and the constructs were verified by direct sequencing. FLAG-tagged JIP1 and JIP3 for expression in mammalian cells were obtained from Addgene (USA) as clones 52123 and 53458, respectively (RRID: Addgene_52123, Addgene_53458).

### Protein expression and purification

Proteins were expressed in the *E. coli* BL21(DE3) strain (NCBITaxon_469008). Briefly, single colonies were picked and grown at 37 °C overnight. Small-scale overnight bacterial cultures grown at 28 °C were used to inoculate 8 × 0.5 L cultures that were incubated at 37 °C until they reached an OD600 of 0.4–0.6. The temperature was then lowered to 18 °C and protein synthesis was induced by the addition of 500 μM isopropyl β-D-1-thiogalactopyranoside (IPTG) for 16 h. Cells were harvested by centrifugation at 5000 *g* for 15 min at 4 °C and resuspended in 50 mM 4-(2-hydroxyethyl)1-piperazineethanesulfonic acid (HEPES) buffer at pH 7.5, 500 mM NaCl, 5 mM b-mercaptoethanol supplemented with protease inhibitor cocktail (Roche, Switzerland; cat. No. 11836170001) and 5 U/ml of Benzonase endonuclease (Merck, Germany; cat. No. 70664). Cell lysis was accomplished by sonication. Insoluble material was sedimented by centrifugation at 16,500 *g* for 1hr at 4 °C and the supernatant microfiltered using a 0.22 mm pore size filter prior to loading on a HisTrap column (GE Healthcare, USA; former RRID:SCR_000004, cat. No. 175248) pre-equilibrated with lysis buffer for immobilised metal affinity chromatography (IMAC). Proteins were eluted with an imidazole gradient and fractions containing the target protein collected and dialysed overnight at 4 °C against imidazole-free lysis buffer. The purification tag was cleaved by incubating the dialysed sample for approximately 5 h at room temperature in the presence of thrombin protease covalently bound to agarose beads (Thrombin CleanCleave Kit, Sigma, USA; RRID:SCR_008988, cat. No. RECOMT). Beads were removed by filtration over a gravitational column and the eluate further microfiltered using 0.22 mm pore size filter prior to loading it on a HisTrap column (GE Healthcare; former RRID:SCR_000004, cat. No. 175248) pre-equilibrated with lysis buffer. Untagged material present in the flow-through fraction was collected, concentrated, and further purified by size exclusion chromatography (SEC) on a 16/60 HiLoad Superdex 75 column (GE Healthcare; former RRID:SCR_000004, cat. No. 28989333) equilibrated with 25 mM HEPES, pH 7.5, 150 mM NaCl, and 5 mM β-mercaptoethanol. Nanobodies were expressed in *E. coli* WK6 cells (ATCC n.47078, NCBITaxon_562). Protein expression in TB medium was induced by addition of 1 mM IPTG at an OD600 of 0.9–1.0, and cells were grown overnight at 28 °C. The periplasm fraction was harvested by osmotic shock, and nanobodies were purified by IMAC and SEC using a HiLoad Superdex 75 column (GE Healthcare; former RRID:SCR_000004, cat. No. 28989333). The Nb:KLC1^extTPR^-JIP1^C-term^ complex used for crystallisation was obtained by mixing the individual components with a 1:1 M ratio and allowing incubation on ice for 30 min. The complex was further purified by SEC on a 16/60 HiLoad Superdex 75 column (GE Healthcare; former RRID:SCR_000004, cat. No. 28989333).

### Differential scanning fluorimetry

Purified proteins and SYPRO orange (Life Technologies, USA; RRID:SCR_008817, cat. n. S6650) were mixed to the final concentration of 2, 5, 10, 30 μM and 8×, respectively, and 25 μl per sample were pipetted into a well of a MicroAmpTM Fast Optical 48-Well Reaction Plates (Applied Biosystems, USA; RRID:SCR_005039, cat. No. 4375816) sealed using MicroAmpTM Optical Adhesive Film (Applied Biosystems; RRID:SCR_005039, cat. No. 4311971). The plate was loaded into a StepOne Real-Time PCR System (Applied Biosystems; RRID:SCR_023455) and the preset “Melting Curve” protocol was run (temperature “step and hold” ramp from 25 to 95 °C at a rate of 0.5 °C/45” with excitation at 470 nm and emission recorded at 610 nm). The data was exported and the melting temperature (T_m_) was calculated in Prism 10.0 (GraphPad; RRID:SCR_002798) by fitting to the Boltzmann equation. Statistical analysis was performed on technical replicates (n = 4) from a single representative highly pure protein variant.

### X-ray crystallography

Crystallisation was performed using the vapour diffusion setup at 18 °C and a 2:1 protein:precipitant ratio in 400 nl sitting drops dispensed with the aid of Mosquito crystallisation robot (TTP LabTech, UK; RRID: SCR_020864). Crystals of the Nb:KLC1^extTPR^-JIP1^C-term^ complex grew from a protein solution concentrated at ∼10 mg/ml in the presence of 0.1 M MMT buffer and 25% polyethylene glycol 1500. For data collection, crystals were cryo-protected using 25% (v/v) ethylene glycol. A dataset at the 2.13-Ǻ resolution was measured at the I03 beam line of Diamond Light Source (UK). The complex crystallised in the monoclinic space group *C*2 with one molecule of the complex in the asymmetric unit. Data were processed using the autoPROC-STARANISO pipeline^33^ (RRID:SCR_015748, RRID:SCR_018362) and the structure was solved by the molecular replacement technique using the software package Phaser^34^ (RRID:SCR_014219). Crystallographic refinement was performed using autoBUSTER (RRID:SCR_015653) and Refmac5^35^ (RRID:SCR_014225). Model building was performed with the molecular graphics package COOT^36^ (RRID:SCR_014222). A summary of data collection and refinement statistics are shown in Supplementary Table S1. Structural images were prepared with PyMol (Schrödinger; RRID:SCR_000305).

### Fluorescence polarisation (FP) measurements

N-terminal TAMRA-conjugated peptides (>95% pure) were supplied either by Tsingke Biotech or synthesised in-house using solid-phase peptide synthesis employing a multiple peptide synthesiser (Syro II, Biotage, Sweden; RRID:SCR_025775). Peptides were purified by reverse-phase high-performance liquid chromatography and their quality confirmed by mass spectrometry. Sequences were as follows: JIP1^C-term^, YTCPTEDIYLE; TorsinA^C-term^, TVFTKLDYYLDD; SKIP^WD^, FNSVTSTNLEWDDSAI; KinTag, GTVFTTEDIYEWDDSAI. FP measurements were performed at room temperature using a Tecan Spark microplate reader (excitation: λ = 535 nm with 25 nm bandwidth; emission: λ = 595 nm with a 35 nm bandwidth; RRID:SCR_021897) by incubating 150 nM TAMRA-labelled peptides dissolved in DMSO with the different KCL1^extTPR^ variants at increasing concentrations (25 mM HEPES, pH 7.5, 150 mM NaCl, 5 mM 2-mercaptoethanol). The final DMSO concentration in the assay buffer was 0.015% (v/v). Samples (30 μl) were dispensed into black non-binding 384-well microplates (Greiner, Austria; RRID:SCR_028044, cat. No. 781900). Estimation of equilibrium dissociation constant (*K*_D_) values was performed assuming a one-site specific-binding model using the Prism 10.0 package (GraphPad Software; RRID:SCR_002798). Data points represented the mean of three replicates with bars representing the standard error.

### Immunoprecipitation

Immunoprecipitation of FLAG-tagged JIP1 and JIP3 and HA-tagged KLC1^TPR^ proteins were essentially performed as previously described.^17^ HeLa cells were transfected with vectors expressing FLAG-JIP1, FLAG-JIP3, and HA-KLC1^TPR^, as well as KLC1^TPR^ containing the patient-derived variants p.(Asp253Ala), p.(Asp253Gly), p.(Met257Val), p.(Ser389Phe), and p.(Leu470Phe). For details, see Supplementary Methods.

### Ethics

The study was performed within the framework of gene-discovery initiatives for rare diseases which has ethical approval from the appropriate review board of Technical University of Munich (Munich, Germany; main reference numbers = 208/21S, 589/20S, and 5360/12S). Additional individuals were identified in different centres worldwide in diagnostic or research settings approved by the respective institutional review boards. All affected individuals or their legal representatives gave written informed consent for the sequencing procedures and the publication of their results along with clinical and molecular data. Data was de-identified. This study adhered to the World Medical Association Declaration of Helsinki (2013). Explicit consent was obtained to publish patient MR images and to include the videos from the participants shown.

### Statistical analysis

The burden of *de novo* missense variants in *KLC1* was evaluated using the R package denovolyzeR (v0.2.0),^37^ which implements a gene-specific mutational model based on sequence context.^38^ In total, 7 *de novo* missense variants in *KLC1* were identified. Formal statistical analysis focused on the 3 variants identified within an unselected cohort of 4700 trios from a single participating sequencing centre (Munich, Germany), comprising all consecutive trio exomes and genomes processed at that site, as this represented the only sub-cohort for which a reliable denominator^37^ was available. The observed variant count was compared against the model-derived expectation using a Poisson test as implemented in denovolyzeR.^37^ Thermal-stability analysis included statistical testing of T_m_ values using two-way ANOVA followed by Dunnett’s multiple comparison. In immunoprecipitation studies, mean differences relative to wild type and their 95% confidence intervals were obtained using one-way ANOVA with Dunnett’s multiple comparison.

### Role of the funding source

The funders of the study had no role in study design, data collection, data analyses, data interpretation, or writing of the report. The corresponding author had full access to all the data in the study and had final responsibility for the decision to submit for publication.

## Results

### Trio sequencing results and identification of *KLC1 de novo* variants

**Fig. 1:**
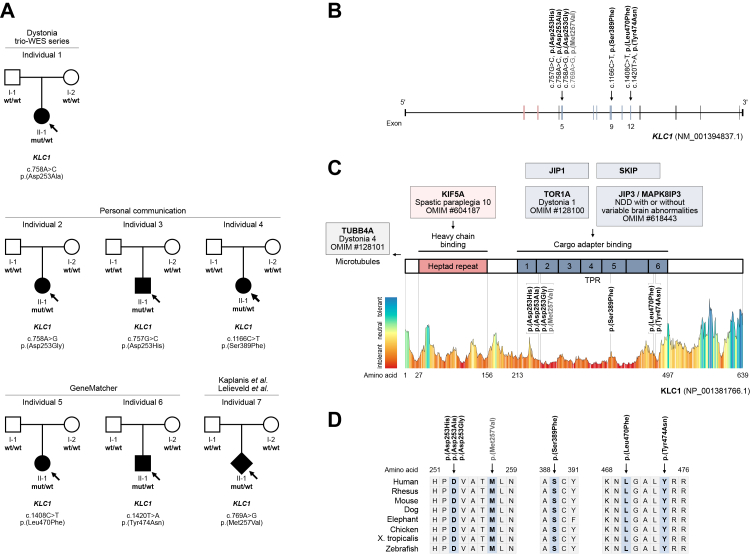
Identification of 7 unrelated individuals with spatially clustering, *de novo KLC1* missense variants. (A) Simplified pedigrees showing the *de novo* status of a distinct *KLC1* missense variant for each identified patient. Asp253 was recurrently mutated in three unrelated affected subjects (individuals 1–3). Unfilled shapes represent unaffected parents while filled-black shapes represent the patients; circles are females, and squares are males. Sex was unknown for individual 7, published as part of a large neurodevelopmental disorder (NDD)/intellectual disability cohort.^1^^,^^2^^,^^28^ Individual 1 was identified in the dystonia trio whole-exome sequencing (WES) series,^7^^,^^8^ while the other individuals were identified via different matchmaking strategies, as indicated above the pedigree drawings.^1^^,^^2^^,^^27^^,^^29^^,^^30^ (B) Linear schematic representation of KLC1 (NM_001394837.1) with indication of identified variants and (C) missense variant clustering in the tetratricopeptide repeat (TPR) domain. Black variants indicate variants detected in the dystonia trio-WES series as well as variants identified through matchmaking via personal communication^29^^,^^30^ and GeneMatcher^27^; the grey variant represents a variant identified from a large published catalogue of *de novo* variants in NDD/intellectual disability.^1^^,^^2^^,^^28^ The MetaDome web server^26^ was used to predict the variation-intolerant amino-acid positions in the TPR domain. All *de novo* KLC1 missense substitutions reported in patients in this study are located at sites depleted of variation in the general population. The TPR domain is highlighted in blue, and the heptad repeat domain required for interaction with the heavy-chain subunit KIF5A is shown in red.^17^ Key interactors of the TPR domain are indicated, alongside their known associations to monogenic neurological diseases.^14^ (D) Amino acid conservation across different species for the 7 variants described in the present work.

We obtained a set of 33 *de novo* non-synonymous variants passing IGV inspection within 32 variation-constrained genes^13^ not yet associated with disease^14^ in 30 index patients from the dystonia trios (30/257; 11.7%; Supplementary Fig. S1B), underlining the potential for discovering yet-undescribed *de novo* causation; all *de novo* variants are detailed in Supplementary Table S2 Of all prioritised *de novo* variants, 26 were predicted missense SNVs in genes with *Z* scores between 3.21 and 5.54, and 7 were SNVs (*n* = 3) and indels (*n* = 4) predicted to cause a loss of function in genes with LOEUF scores between 0.596 and 0.28; 75.8% (25/33) of the variants were absent from gnomAD-v4.1.0 and our in-house control databases. For the missense variants, CADD^23^ yielded a mean score of 24.4 (range: 9.7–32.0). We then sought to identify further independent subjects with matching disease presentations who also had *de novo* variants in the constrained genes that were found to be mutated in our dystonia trio-WES series. Among these genes, 31 (31/32) were considered to represent potential candidates pending additional evidence, including 18 genes (18/31) for which follow-up case-matching and/or functional-study efforts are still underway; for details, see Supplementary Table S2. It is important to note that disease relevance has not been established for any of these 31 candidates at present. By contrast, the current identification of *de novo* missense variants in 7 unrelated patients with similar phenotypic characteristics in *KLC1* provided strong candidate evidence for a shared genetic aetiology (Table 1; Table 2; Fig. 1A–D). Individual 1 from the dystonia series harboured the *KLC1 de novo* variant (NM_001394837.1): c.758A>C, p.(Asp253Ala) (Fig. 1A); of note, this individual was originally identified in the context of a large dystonia-exome study,^7^ in which *KLC1* was flagged as a candidate gene following detection of a single *de novo* missense variant among more than 700 sequenced families with dystonia. Through direct personal communications within research networks^29^^,^^30^ and data submission to GeneMatcher,^27^ we ascertained 2 more patients with *KLC1 de novo* variants affecting the same amino-acid position, c.758A>G, p.(Asp253Gly) (individual 2) and c.757G>C, p.(Asp253His) (individual 3), as well as 3 additional patients with *de novo* variants in *KLC1*, including one with c.1166C>T, p.(Ser389Phe) (found in probable mosaic state, ∼23% in blood DNA, 9/39 reads; individual 4), one with c.1408C>T, p.(Leu470Phe) (individual 5), and one with c.1420 T>A, p.(Tyr474Asn) (individual 6). Another *KLC1 de novo* variant, c.769 A>G, p.(Met257Val) carried by individual 7, has been previously reported but without characterisation of its molecular features^1^^,^^2^^,^^28^ (Fig. 1A). The mosaic status of c.1166C>T, p.(Ser389Phe) was confirmed by Sanger sequencing of blood-derived DNA from individual 4, although it was not possible to assess variant allele fraction across additional tissues. The 7 different *KLC1* variants affected conserved nucleotides and residues and were predicted to be deleterious by most applied bioinformatic tools (Table 1; Fig. 1B and D). MetaDome analysis^26^ indicated that each variant position was (highly) intolerant to alterations at the amino-acid level (Fig. 1C). *In silico* predictions were considered as supporting evidence, consistent with ACMG PP3 criteria^4^ (Table 1); no single predictor was used as primary evidence for pathogenicity classification (Table 1). None of the variants were observed in gnomAD-v4.1.0 or in-house reference datasets. Structurally, KLC1 consists of a N-terminal coiled-coil heptad repeat domain that interacts with the heavy-chain subunit, followed by an unstructured linker region that connects to a TPR domain linking kinesin-1 to many of its cargos.^17^ The TPR domain is followed by a carboxy-terminal domain that varies between splicing isoforms. All 7 detected variants mapped on KLC1`s solenoid-shaped TPR domain (Fig. 1B and C; Supplementary Fig. S3), constituted of 6 helix-turn-helix TPR units with TPR5 and TPR6 connected by a non-TPR helix (αN) located within a flexible region. Asp253 and Met257 are situated on the first half of α3 of TPR2. Ser389 is located on α9 of TPR5 with its side-chain solvent exposed on the domain’s concave surface. Leu470 and Tyr474 situated on α11 of TPR6 are more buried^17^ (Supplementary Fig. S3). To formally evaluate the statistical significance of *KLC1 de novo* variant enrichment, we performed burden testing using denovolyzeR,^37^ focusing the analysis on the 3 variants ascertained from an unselected single-centre cohort of 4700 trios sequenced in Munich, Germany. Under the background mutational model,^38^ 0.17 *de novo* missense variants would be expected in *KLC1* across this cohort size. The observed count of 3 represented a 17.2-fold enrichment (Poisson test, p = 7.8 × 10^−4^), providing formal statistical support for a non-random accumulation of *KLC1 de novo* missense variants. The identification of 4 additional *de novo* missense variants in *KLC1* from independent international cohorts further strengthens this observation, although these could not be incorporated into the formal burden analysis owing to the absence of cohort-level denominators for those datasets.

**Table 1:** Overview of rare heterozygous *de novo KLC1* missense variants in seven individuals with movement and neurodevelopmental disorders.

Individual	1	2	3	4	5	6	7 (Kaplanis et al., PMID: 33057194; Lelieveld et al., PMID: 27479843)
*KLC1* variant RefSeq transcript: NM_001394837.1	c.758A>C, p.(Asp253Ala)	c.758A>G, p.(Asp253Gly)	c.757G>C, p.(Asp253His)	c.1166C>T, p.(Ser389Phe)^a^	c.1408C>T, p.(Leu470Phe)	c.1420 T>A, p.(Tyr474Asn)	c.769 A>G, p.(Met257Val)
Chromosomal position (GRCh37/hg19)	chr14:104129225A>C	chr14:104129225A>G	chr14:104129224G>C	chr14:104139673C>T	chr14:104143780C>T	chr14:104143792 T>A	chr14:104129236 A>G
Inheritance	*de novo*	*de novo*	*de novo*	*de novo*	*de novo*	*de novo*	*de novo*
Frequency in-house sequencing datasets (>30,000)	not found	not found	not found	not found	not found	not found	not found
Frequency gnomAD v4.1.0	not found	not found	not found	not found	not found	not found	not found
Conservation (GERP)	5.19	5.19	5.19	4.83	5.52	5.52	5.19
CADD GRCh37-v1.7	28.2	27.3	32	32	28.7	27.1	24.9
REVEL	0.61	0.7	0.58	0.59	0.68	0.78	0.49
AlphaMissense	0.999	1	1	0.995	0.999	0.999	0.99
MetaDome	variation-intolerant amino acid residue	variation-intolerant amino acid residue	variation-intolerant amino acid residue	variation-intolerant amino acid residue	variation-intolerant amino acid residue	variation-intolerant amino acid residue	highly variation-intolerant amino acid residue
Localisation within encoded protein	TPR domain	TPR domain	TPR domain	TPR domain	TPR domain	TPR domain	TPR domain
Applicable ACMG criteria	PS2, PS3, PM2, PP3	PS2, PS3, PM2, PP3	PS2, PM2, PP3	PS2 (mosaic), PS3, PM2, PP3	PS2, PM2, PP3	PS2, PM2, PP3	PS2, PS3, PM2, PP3

ACMG, American College of Medical Genetics and Genomics; CADD, Combined Annotation Dependent Depletion; GERP, Genomic Evolutionary Rate Profiling; gnomAD, Genome Aggregation Database; REVEL, Rare Exome Variant Ensemble Learner; TPR, tetratricopeptide repeat.

a Mosaic status, ∼23% in blood DNA based on trio WES results (9/39 reads); confirmed by Sanger sequencing of individual 4`s blood DNA.

**Table 2:** Overview of phenotypic findings for seven individuals with rare heterozygous *de novo KLC1* missense variants.

Individual	1	2	3	4	5	6	7 (Kaplanis et al., PMID: 33057194; Lelieveld et al., PMID: 27479843)
Sex/age last evaluated	female/19 y	female/5 y	male/5 y	female/15 y	female/8 y	male/6 y	N/A
Origin (recruitment country)	European (Czechia)	European (USA)	European/African (Germany)	European (Germany)	European (Switzerland)	European (Italy)	N/A (N/A)
Recruited through	dystonia trio-WES series	personal communication (clinical trio WES)	personal communication (clinical trio WGS)	personal communication (research trio WES)	GeneMatcher (research trio WES)	GeneMatcher (research trio WES)	published *de novo* variant catalogue
Working clinical diagnosis	dystonic-spastic cerebral palsy	dystonic-spastic cerebral palsy	dystonic-spastic cerebral palsy	neuromuscular disorder/contractures	neurodevelopmental disorder	neurodevelopmental disorder	neurodevelopmental disorder^a^
Similarly affected family members including siblings where present	–	–	–	nr	–	–	N/A
HPO terms for affected subject	HP:0001332, HP:0001257, HP:0001253, HP:0001252, HP:0001263, HP:0001249, HP:0002364, HP:0002572, HP:0002015, HP:0011968, HP:0002007, HP:0000455, HP:0002500, HP:0002120	HP:0001332, HP:0001257, HP:0001253, HP:0001252, HP:0001263, HP:0001249, HP:0002364, HP:0002572, HP:0002015, HP:0002360, HP:0004209, HP:0002079, HP:0002500	HP:0001332, HP:0001257, HP:0001253, HP:0001252, HP:0001263, HP:0001249, HP:0002364, HP:0002572, HP:0001250, HP:0000369, HP:0000639, HP:0002188, HP:0007109	HP:0001252, HP:0001257, HP:0001371	HP:0001252, HP:0001263, HP:0001249, HP:0000750, HP:0012758, HP:0000256, HP:0000733	HP:0001263, HP:0001249, HP:0000750, HP:0000729, HP:0003212	HP:0001249, HP:0001263
Movement disorder(s); age at onset; first affected body part	dystonia, spasticity; ∼6 months; leg	dystonia, spasticity; ∼6 months; leg	dystonia, spasticity; ∼8 months; leg	increased tone; since childhood; leg	-; N/A	-; N/A	N/A
Hypotonia in infancy	+	+	+	+	+	–	N/A
Developmental delay	+	+	+	nr	+	+	N/A
Intellectual impairment	+	+	+	nr	+	+	+^a^
Behavioural deficits	nr	nr	nr	nr	+ (significant behavioural rigidity, lack of reciprocity in exchanges)	+ (autism spectrum disorder)	N/A
Speech impaired	+	+	+	nr	+	+	N/A
Seizures	–	–	+ (focal seizures, history of status epilepticus)	–	–	–	N/A
Dysmorphic features	+ (broad forehead, bulbous nasal tip)	nr	+ (posteriorly rotated ears)	nr	–	nr	N/A
Other reported relevant abnormalities	+ (dysphagia, feeding difficulties, ophistotonus)	+ (dysphagia, ophistotonus, insomnia, bilateral fifth digit clinodactyly)	+ (ophistotonus, fine-beating nystagmus)	nr	+ (relative macrocephaly)	+ (high IgE levels)	N/A
Brain MRI abnormalities	+ (mild cortical atrophy, white matter loss)	+ (thinning of corpus callosum, mild cerebral white matter volume loss)	+ (confluent T2 signal hyperintensities of the periventricular white matter, delayed myelination)	N/A	N/A	N/A	N/A

+, feature present; –, feature not present.

HPO, Human Phenotype Ontology; MRI, magnetic resonance imaging; N/A, not available or not applicable; nr, not reported; WES, whole-exome sequencing; WGS, whole-genome sequencing; y, years.

a No detailed phenotypic information available for individual 7 from published large trio analysis cohorts.

### Phenotypic spectrum associated with *KLC1* variants

We were able to collect unpublished clinical data for the individuals with *KLC1 de novo* variants, with the exception of individual 7 from the large reported cohort^1^^,^^2^^,^^28^ who was more broadly labelled as having neurodevelopmental disorder/intellectual disability. The patient group consisted of 4 females and 2 males, aged between 5 and 19 years (data available in 6/7; Table 2). The clinical presentations across the 7 individuals were heterogeneous overall; motor dysfunction and/or neurodevelopmental impairment were present across the series, but these features did not co-occur in all individuals, reflecting phenotypic variability. Individuals 1–3 in whom Asp253 was recurrently affected by 3 different missense substitutions were diagnosed with infantile-onset (∼6–8 months) movement disorders with prominent dystonia and spasticity; their conditions were classified as dystonic-spastic cerebral palsy, and the first affected body part was the lower extremity in all 3 individuals (Table 2) These individuals presented consistent courses with delayed motor development, hypotonia evolving into generalised dystonia combined with 4-limb spastic tone (legs > arms), and speech impairment with pronounced articulation problems. Ophistotonic trunk posturing was a recurrent sign in the subjects (3/3, Table 2); they also had intellectual disability, pseudobulbar syndromes with dysphagia and feeding difficulties, and one patient (individual 3) experienced seizure activity. Individual 4 presented with a neuromuscular disorder with muscle tone abnormalities predominantly affecting the proximal extremities, which were clinically interpreted as contractures of shoulder joints and knees. Individuals 5 and 6 exhibited global developmental delay, intellectual disability ranging from mild to moderate severity, speech abnormalities, and behavioural disturbances with significant rigidity (individual 5) and various autistic-type expressions (individual 6). Other variable anomalies in the group included insomnia (individual 2), relative macrocephaly (individual 5), and hyper-IgE syndrome (individual 6). There was no apparent consistent facial dysmorphism despite the presence of subtle aspects, such as a broad forehead and bulbous nasal tip in individual 1 and abnormally set ears in individual 3. Brain-MRI screening was performed for 3 individuals (Table 2; Supplementary Fig. S4) and showed variable neuroanatomical changes, including mild cortical atrophy and associated white matter loss in individual 1, thinning of the corpus callosum in individual 2, and T2 signal hyperintensities of the periventricular white matter with myelination defects in individual 3. Collectively, the clinical spectrum was heterogeneous, although similar patterns seemed to be present according to variant locations, with predominant motor dysfunction and other accompanying neurodevelopmental impairment for variants in the TPR2 and TPR5 motifs (as far as data were available) and more unspecific intellectual-disability syndromes without movement disorders for variants in the TPR6 motif (Table 1; Table 2; Fig. 1B and C).

### Characterisation of *KLC1* variant effects

#### Solubility and stability of KLC1 protein variants

**Fig. 2:**
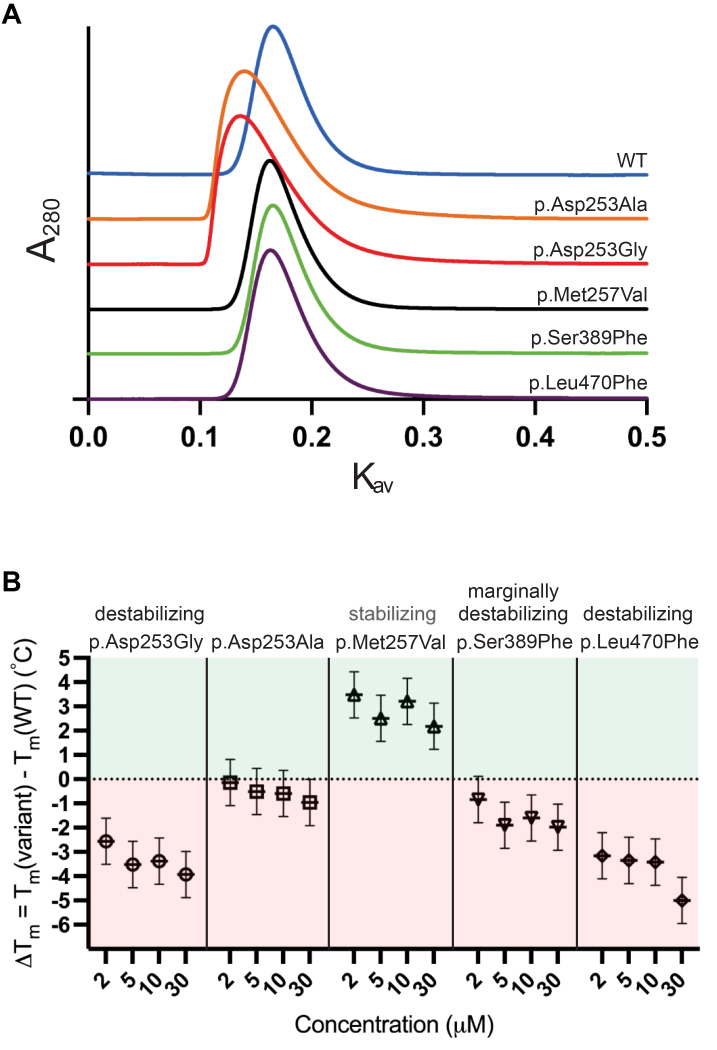
Analysis of solubility and melting temperatures for KLC1 missense variants. (A) Size exclusion chromatography (SEC) elution profiles of purified KLC1^extTPR^ proteins. Normalised absorption at 280 nm (A_280_) is plotted as function of K_av_ = (V_e_-V_0_)/(V_t_-V_0_), where V_e_ = elution volume, V_0_ = void volume, and V_t_ = column volume. All samples eluted as monomers although the p.(Asp253Ala) and p.(Asp253Gly) variants exhibited a smaller K_av_. WT, wild type. (B) Difference in melting temperature (ΔT_m_) values between variants and wild type (WT) KLC1^extTPR^ at the 4 concentrations tested. Error bars correspond to 95% confidence intervals. Statistical analysis was performed on technical replicates (n = 4). Differences between means were analysed by two-way ANOVA followed by Dunnett’s multiple comparisons test. Further information on T_m_ values and statistical analysis is provided in Supplementary Tables S3 and S4.

To evaluate the possible impact of patient-specific KLC1 amino-acid variations on protein folding, we bacterially expressed and purified to homogeneity wild-type KLC1^extTPR^ (residues 172–496), as well as 5 representative patient variants selected to cover the key affected structural regions of the TPR domain: p.(Asp253Ala), p.(Asp253Gly), p.(Met257Val), p.(Ser389Phe), and p.(Leu470Phe). All constructs were soluble, and SEC analysis demonstrated that they eluted as monomers. We noted, however, that the p.(Asp253Ala) and p.(Asp253Gly) variants eluted marginally earlier than all other samples (Fig. 2A), suggesting a larger hydrodynamic radius. We next tested the thermal stability of all constructs using differential scanning fluorimetry at multiple protein concentrations (2–30 μM range) (Fig. 2B; Supplementary Tables S3 and S4). All samples displayed average T_m_ values within the range common for medium-T_m_ proteins of the mouse or human genome^39^ with the wild-type protein unfolding at an average of 55.62 ± 0.39 °C at 5 μM. Statistical analysis of T_m_ values is shown in Supplementary Table S4. Generally, all proteins displayed a mild concentration dependence with the highest T_m_ at 5 μM, which progressively decreased at higher concentrations (Supplementary Table S3). As shown in Fig. 2B, while the p.(Asp253Gly), p.(Ser389Phe), and p.(Leu470Phe) variants showed a consistent reduction in thermal stability with ΔT_m_ values ranging from −0.8 °C to −5.0 °C relative to the wild-type protein, the near-WT stability of p.(Asp253Ala) indicated that the protein scaffold was sensitive to side-chain geometry and flexibility beyond just the loss of the native charge, whereas the p.(Met257Val) variant was the sole construct to exhibit enhanced thermal robustness (+2.2–3.5 °C), likely due to optimised hydrophobic packing.

#### Crystallographic and binding-affinity studies

**Fig. 3:**
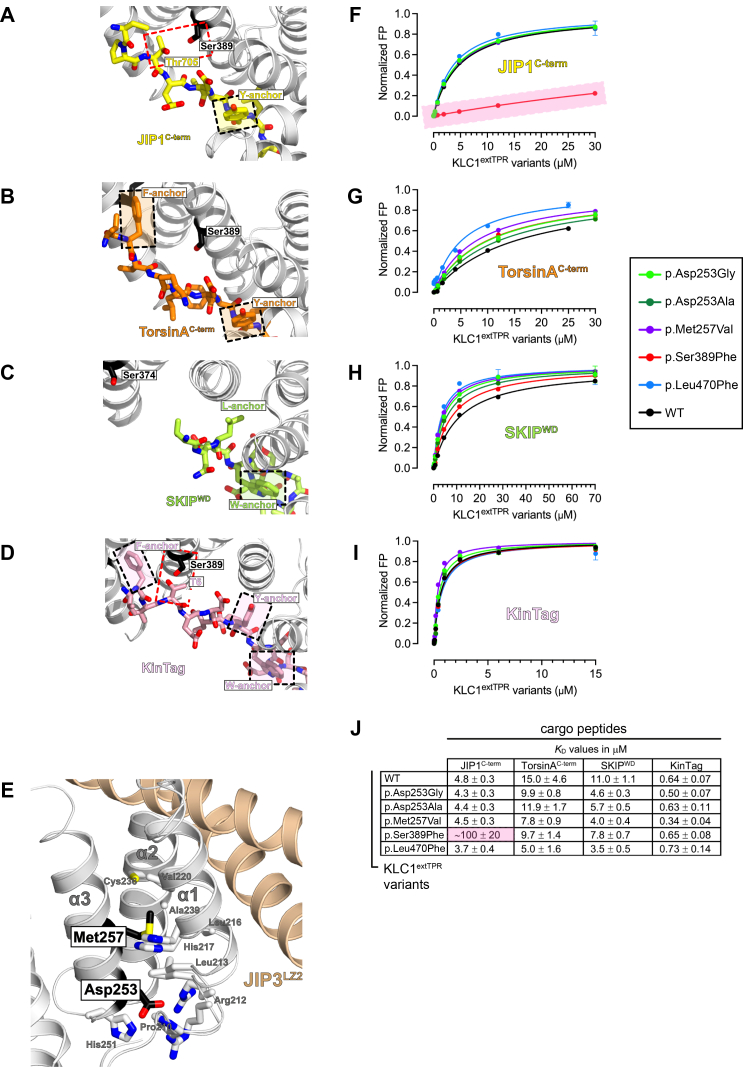
Binding affinities of KLC1 missense variants for different cargo SLiMs. (A,B,C,D) Cartoon representations of KLC1^TPR^ bound to JIP1^C-term^ (A), KLC1^TPR^ bound to TorsinA^C-term^ (B), KLC2^TPR^ bound to SKIP^WD^ (C), and KLC1^TPR^ bound to KinTag (D). Short linear motifs (SLiMs)’ ‘anchoring’ residues that bind within the tetratricopeptide repeat (TPR) domain groves are highlighted by coloured boxes. The close proximity of Ser389 with Thr705 and Thr6 of JIP1 and KinTag, respectively, is highlighted by red rectangles. Residue Ser374 in the KLC2^TPR^-SKIP^WD^ complex (C) corresponds to Ser389 in KLC1^TPR^. (E) Cartoon representation of the N-terminal portion of the KLC1^TPR^-JIP1^C-term^ complex with the α1 helix of TPR1 modelled in double conformation. The location of the JIP3^LZ2^ parallel coiled coil interacting with TPR1 (modelled from PDB: 6EJN) is also shown. The side chains of residues Asp253 and Met257 are highlighted in black with nearby residues shown in grey. (F, G, H, I) Fluorescence polarisation (FP) measurements for wild-type KLC1^extTPR^ and its patient-derived variants with JIP1^C-term^ (F), TorsinA^C-term^ (G), SKIP^WD^ (H), and KinTag (I). Data points have been measured in triplicate and are reported with their standard deviation. Fitting has been carried out in Prism (GraphPad) using the one-site specific model. WT, wild type. (J) Summary of binding affinities from FP measurements. The p.(Ser389Phe) variant selectively disrupts JIP1 binding. WT, wild type.

Cargo binding to KLC1 is mainly accomplished through the interaction of its TPR domain with adaptor molecules. Structurally characterised interactions include the binding of the internal W-acidic and C-terminal Y-acidic short linear motifs (SLiMs) in SKIP and JIP1, respectively, to the concave grove of the TPR solenoid.^17^^,^^40^ The dystonia-related protein TorsinA has also been shown to behave as an adaptor and/or cargo-anchoring modifier of KLC1 by interacting with the TPR domain, in a Y-acidic-like fashion.^17^ Furthermore, an engineered SLiM dubbed ‘KinTag’ which fuses selected elements of the TorsinA, JIP1, and SKIP motifs binds KLC1^TPR^ and KLC2^TPR^ with higher affinity than its parent motifs and when genetically encoded, it promotes transport of lysosomes with higher efficiency than the natural sequences.^41^ Although all structurally characterised adaptor SLiMs bind to the concave surface of the TPR domain, sequence variations around key conserved residues dictate positional and conformational specificities. As part of this work, we solved the crystal structure of the KLC1^extTPR^–JIP1^C-term^ interaction at a higher resolution (2.13 Å) than the previously deposited structure (2.70 Å, PDB: 6FUZ); see Supplementary Table S1 for details. This higher resolution allowed for a more accurate description of key interactions with this adaptor SLiM. Based on crystallographic information, we predicted that the p.(Ser389Phe) replacement would have a significant impact on binding with the Y-acidic JIP1^C-term^, as residue Ser389 is in close proximity to JIP1 Thr705 (Fig. 3A). A phenylalanine at this position was expected to result in steric clashes and disrupt stabilising water-mediated hydrogen bonds. Although TorsinA^C-term^ binds similarly to JIP1^C-term^, sequence differences result in their Y-acidic SLiMs adopting distinct conformations at their N-terminal binding regions. The TorsinA interaction was expected to be more permissive to the p.(Ser389Phe) replacement than the JIP1 interaction, as in addition to the common Y-anchor, the former is also stabilised by its “F-anchor” (Fig. 3B). In contrast, the W-acidic motif of SKIP binds more N-terminally on the TPR scaffold (Fig. 3C). The engineered KinTag features a Thr residue at the same position as JIP1^C-term^, making it potentially sensitive to the p.(Ser389Phe) replacement; however, it also displays multiple F-, Y-, and W-anchoring points that provide compensatory interactions (Fig. 3D). All other patient amino-acid variations mapped far away from the SLiMs-binding region. Residues Asp253 and Met257 were located at beginning of helix α3 of TPR2, adjacent to helix α1 of TPR1 (Supplementary Fig. S3). TPR1 is functionally important as it is involved in mediating the interaction with the coiled-coil/leucine zipper LZ2 of JIP3.^42^ More recently, TPR1 has been shown to interact with a portion of the kinesin-1 heavy chain subunit.^17^ During our here-presented crystallographic refinement of the KLC1-JIP1 complex, it became evident that electron density for the first α-helix of TPR1 was significantly less defined compared to all others with very little density visible for most of its side chains. At the current 2.13 Å resolution, residual difference density suggested that this region is best modelled by two helices translated along the helical axis by approximately 1.5 Å (Fig. 3E). Structural analysis placed Asp253 within interaction distance of the disordered Arg212 side chain on α1-TPR1 and the imidazole group of His251. Consequently, the Asp253 replacements were expected to abolish any existing hydrogen bond or salt–bridge interactions at this interface, likely enhancing local mobility. The side chain of Met257 is surrounded by a mostly hydrophobic cluster of residues: Leu213, Leu216, His217, and Val220 on α1-TPR1 and Cys236 and Ala239 on α2-TPR1 (Fig. 3E). Thus, the p.(Met257Val) replacement preserved local hydrophobicity, with the shorter valine side chain likely changing local packing as indicated by its improved thermal stability. We concluded that p.(Asp253Gly), p.(Asp253Ala), and p.(Met257Val) replacements were unlikely to have a direct effect on SLIMs binding but we hypothesised that they might affect the interaction with JIP3. On the basis of the available structural information, the p.(Leu470Val) replacement was not expected to have direct consequences on the binding of known interactors. We tested our structure-guided predictions in the context of different cargo SLiMs using FP measurements (Fig. 3F-IE-H). As predicted, the p.(Ser389Phe) replacement abrogated JIP1^C-term^ binding, whilst p.(Asp253Ala), p.(Asp253Gly), p.(Met257Val), and p.(Leu470Phe) had limited impact on the binding of the SLiMs tested; a summary of all binding affinities is provided in Fig. 3J.

#### Cell-based molecular interaction analyses

**Fig. 4:**
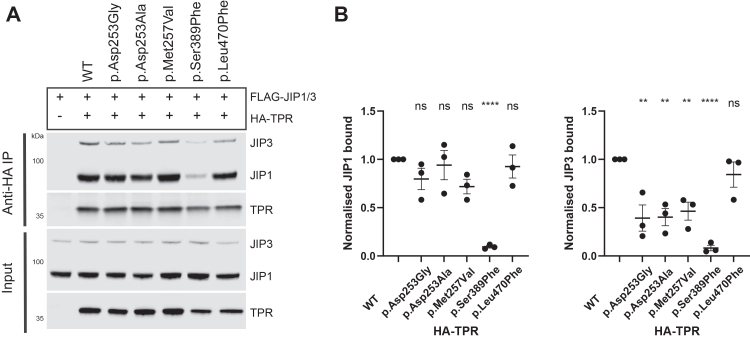
Effect of identified missense substitutions on the formation of the KLC1-JIP1-JIP3 complex. (A) Cell-based assessment of impaired interactions between the tetratricopeptide repeat (TPR) domain of KLC1 and its binding partners JIP1 and JIP3 in the presence of patient-derived *KLC1* missense variants. For immunoprecipitation experiments, HeLa cells were transfected with the indicated HA-TPR, FLAG-JIP1 and FLAG-JIP3 expression constructs, lysed and immunoprecipitated using anti-HA-agarose. Bound proteins (top) and expression in input cell extracts (bottom) were analysed by Western blot using anti-HA and anti-FLAG antibodies. WT, wild type. (B) Quantifications from (A) from 3 independent experiments following normalisation to input expression levels. For JIP1 (see also Supplementary Table S5), mean differences relative to wild type (WT) and their 95% confidence intervals (in brackets), obtained using one-way ANOVA with Dunnett’s multiple comparisons test, were: Asp253Gly 0.20 (−0.19 to 0.60), Asp253Ala 0.06 (−0.33 to 0.45), Met257Val 0.28 (−0.11 to 0.68), Ser389Phe 0.91 (0.51–1.30), and Leu470Phe 0.07 (−0.32 to 0.47). For JIP3 (see also Supplementary Table S5), mean differences relative to wild type (WT) and their 95% confidence intervals, obtained using one-way ANOVA with Dunnett’s multiple comparisons test, were: Asp253Gly 0.61 (0.22–0.99), Asp253Ala 0.60 (0.21–0.98), Met257Val 0.54 (0.15–0.92), Ser389Phe 0.92 (0.53–1.30), and Leu470Phe 0.16 (−0.23 to 0.54). Error bars show ± SEM. ∗∗∗∗ = p < 0.0001, ∗∗ = p < 0.01, ns = not significant.

To investigate how patient-derived variants affect cargo recruitment in a cellular environment we performed immunoprecipitation studies (Fig. 4A and B; Supplementary Fig. S5 and Table S5). HeLa cells exhibited markedly reduced binding of KLC1^TPR^ containing p.(Ser389Phe) to both JIP1 and JIP3 compared to wild-type, confirming and expanding upon our predictions and FP assays. Furthermore, when accounting for expression levels, we determined that binding of KLC1^TPR^ to JIP3 was reduced to a statistically significant extent in cells expressing the p.(Asp253Ala), p.(Asp253Gly), and p.(Met257Val) variants, but not p.(Leu470Phe). Together, these findings demonstrated that whilst the p.(Ser389Phe) variant disrupted JIP1 binding resulting presumably in a diminished JIP3 recruitment, the more N-terminal p.(Asp253Ala), p.(Asp253Gly), and p.(Met257Val) variants impacted directly on JIP3 binding. Thus, all the variants tested except for p.(Leu470Phe) perturbed the cargo-adaptor binding properties of the KLC1^TPR^ domain in the context of the KLC1-JIP1-JIP3 functional ternary complex.

## Discussion

Herein, we report on a survey of candidate *de novo* variants affecting genes with significant constraint in the general population^13^ that were found among individuals with dystonia. These data are made available to the community for ongoing matchmaking and future gene–phenotype association research.

We successfully assembled a set of disease subjects who each harboured a *de novo* missense variant in *KLC1*, a member of the genes encoding KIFs. These motor proteins exhibit wide expression throughout the brain, consistent with their importance in orchestrating complex intracellular transport systems that are vital to neural development and survival.^11^^,^^12^ Notably, *KLC1* had previously emerged as a candidate gene in our 2020 dystonia-exome study.^7^ Only one *KLC1* variant was identified across more than 700 families in that study,^7^ suggesting that *KLC1*-related disease represents a rare cause of dystonic syndromes.

Data from animal models have contributed to the definition of a key neurodevelopmental role for *KLC1*. Mutant mice lacking normal *Klc1* demonstrated marked movement abnormalities on phenotypical analyses and their sensory and motor neurons were found to display abnormal localisation and functionality of kinesin-1^43^; it has thus been postulated that other Klc paralogues cannot compensate for defective *Klc1*.^43^ Moreover, a pronounced neuronal phenotype with paralysis, pathological aggregates reflective of non-transported cargos in axonal structures, as well as early lethality have been described for *Klc*-deficient *Drosophila* melanogaster.^44^

Our data represent compelling multi-layered evidence implicating specific *KLC1* variants in the causation of movement and neurodevelopmental disorders. Converging lines of evidence from our genomic and experimental explorations are consistent with pathogenicity for a previously undescribed monogenic entity related to *KLC1*: (1) each variant occurred *de novo* in affected individuals; (2) all variants were unobserved in large population-control datasets, while an identical amino-acid residue (Asp253) was altered in 3 unrelated patients; (3) the mutated amino acids displayed high phylogenetic conservation and mapped to the same essential protein domain; (4) *in silico* analyses predicted putative deleteriousness for the variants; and (5) the variants that we tested functionally resulted in varying degrees of KLC1 stability changes and/or disturbed cargo-adaptor binding capacity. Although the phenotypic spectra presented by the 7 identified individuals were variable, it is interesting to note that several direct and indirect interactors of KLC1 are encoded by genes in which rare dominant variants are causative of phenotypes that bear important overlap with the presentations of our patients. The clinical features of *MAPK8IP3* (encoding JIP3)-associated neurodevelopmental disorder with or without variable brain abnormalities (NEDBA; OMIM:618443) encompass psychomotor delay, impaired intellectual development of variable severity, speech disturbances, and motor dysfunction with spasticity and dystonic symptoms^45^, ^46^, ^47^; variants underlying NEDBA were proposed to lead to perturbed binding of JIP3 to KLC family members,^45^ and a dystonia-linked *MAPK8IP3* missense variant was found to disrupt axonal transport with accompanying dopaminergic deficits in patient-derived cell lines.^47^ A recurrent in-frame deletion variant in *TOR1A* (encoding TorsinA) is associated with early-onset generalised dystonia^14^ (DYT-*TOR1A*; OMIM:128100); in cultured cortical neurons, this particular variant impacted on the co-localisation of TorsinA and KLC1, suggesting potential negative effects on kinesin-1 activity.^20^ Individuals with variants in *KIF5A* (encoding KIF5A) typically exhibit spastic-dystonic movement disorders combined with cognitive impairment^14^^,^^21^; HSP-*KIF5A*-causing variants are known to impair cargo transport,^48^ a mechanism that could also be anticipated for the herein-reported KLC1 variants. Furthermore, it was shown that a specific missense alteration in *TUBB4A* (encoding tubulin beta-4A) gives rise to reduced binding of kinesin-1 to microtubules,^49^ providing a mechanistic explanation for the associated phenotype characterised by dystonia, spasticity, and severe neurodevelopment comorbidity^14^ (HSP/DYT-*TUBB4A*; hypomyelinating leukodystrophy-6; OMIM:612438). In aggregate, these observations underline strong phenotypic similarities between monogenic diseases associated with defects of intracellular trafficking along microtubules, including the broader class of ‘kinesinopathies’^50^ caused by pathogenic variants in KIFs. It is plausible that the similarities in disease outcomes could be related to a convergence in pathophysiology (Supplementary Fig. S6), involving impaired functional interactions between mutated KIFs, their adaptors, and/or components of microtubules. Similarly to subjects affected by previously described kinesinopathies,^50^ our individuals 1–4 with KLC1 variants in TPR2/5 motifs experienced pronounced motor abnormalities from early childhood, variably accompanied by other features of impaired neurodevelopment such as cognitive dysfunction and speech disturbances. Two variants located in another motif of KLC1ˋs TPR domain (TPR6) resulted in neurodevelopmental disease-predominant presentations with developmental delay, intellectual disability, and behavioural symptoms, but no overt movement disorder (individuals 5 and 6). Additional work will be necessary to better define genotype–phenotype correlations in *KLC1*-related diseases and to clarify whether the different variant localisations predispose to specific clinical manifestations.

New insights into relationships between human diseases and rare variants in the genes encoding KIFs, such as KLCs, can provide further impetus for the evaluation of their functional importance. Two of the KLC family genes have been proposed to be associated with monogenic syndromes: *KLC2* with autosomal recessive spastic paraplegia, optic atrophy, and neuropathy^14^^,^^51^ (HSP-*KLC2*; OMIM:609541); and *KLC4* with autosomal recessive early-childhood-onset neurodegeneration with retinitis pigmentosa, sensorineural hearing loss, and demyelinating peripheral neuropathy^14^^,^^52^ (OMIM:621129). However, these associations have not been convincingly replicated^51^^,^^52^ and no prior publications have reported the characterisation of the molecular interplay between KLCs and their binding partners in the context of patient-derived variant alleles. A recent dystonia-exome study identified rare *KLC1* missense and non-coding variants^9^; however, none of these were demonstrated to have arisen *de novo*, and no functional characterisation was performed. It therefore remains uncertain whether these reported variants were disease-relevant. Our biophysical, biochemical, and cell-based studies allowed for beginning meaningful inferences about the pathogenic effects of disease-related missense variants in *KLC1*. Our approach of predicting and testing the binding affinity of KLC1ˋs C-terminal portion containing the TPR domain to the established interactors JIP1, JIP3, SKIP, and TorsinA in the presence of patient-derived missense substitutions highlighted potential distinctive mechanisms of pathogenicity for the *de novo* variants. We observed the most severely reduced binding to JIP1 and JIP3 for the p.(Ser389Phe) variant, whereas the variants p.(Asp253Ala), p.(Asp253Gly), and p.(Met257Val) resulted in moderate but statistically significant binding impairment for JIP3. These results may be consistent with a dominant-negative effect. We anticipate that the interplay between KLC1 and its binding partners may be disturbed in a more complex fashion in the majority of our patients, and will not be limited to the adaptor proteins tested here. The KLCs also play important roles in controlling the activity of the kinesin-1 holoenzyme, utilising molecular interfaces that overlap with those used for cargo binding, and therefore pathological consequences from a generalised dysregulation of kinesin-1 activity could also be considered. Nevertheless, the presented data clearly indicate that changes in TPR domain functionality may be important in the pathogenesis of *KLC1*-associated disease phenotypes.

Based on the phenotypes of the patients collected in this study, we cannot distinguish between a pure neurodevelopmental and a co-occurring neurodevelopmental-neurodegenerative pathology.^11^^,^^12^ Proper delivery of axonal cargos controlled by KIFs is crucial for synapse development and the establishment of brain connectivity.^11^ On the other hand, dysfunctional KIFs were shown to promote neurodegeneration, as neurons are particularly reliant on efficient transport processes for maintenance of synaptic homoeostasis.^12^ Future studies in patient-derived neural cells would be useful to define the pathophysiological substrates in *KLC1*-related diseases, which in turn could provide insights into targeted therapeutic interventions. Of note, experimental therapeutics directed to axonal transport are currently under study, such as kinase inhibitors that modulate microtubule modifications^12^ and antisense oligonucleotides that may ameliorate KLC cargo-adaptor dysfunction.^47^

The strengths of this study lie in the integration of orthogonal lines of evidence, including systematic trio-based genomic analysis, international case-matching, formal *de novo* enrichment testing, and multi-assay functional characterisation encompassing structural biology, biophysical binding measurements, and cell-based interaction studies. Limitations should be acknowledged. The total number of reported individuals remains small, restricting formal genotype–phenotype correlation analyses and precluding definitive assessment of the full clinical spectrum. Ascertainment bias cannot be excluded, as case identification through specialty clinics may enrich for more severely affected individuals. Primary patient-derived cell material was not available, hindering direct examination of endogenous KLC1 function in patient-derived fibroblasts, which remains an avenue for future work. The possibility that variants in individuals with less specific neurodevelopmental phenotypes (individuals 5 and 6) represent incidental findings in constrained genes rather than causal events or act in the context of more complex genetic architectures cannot be formally excluded. Finally, *KLC1*-related disorder is expected to be rare in unselected dystonia populations.^7^

In summary, our study indicates that a residual pool of undiagnosed dystonias may be related to ultrarare *de novo* variants in genes that have not yet been associated with monogenic diseases. This is supported by the finding of putatively deleterious *de novo* alterations in variation-constrained genes in ∼12% of unresolved cases in our trio WES series. We further provide evidence that heterozygous *de novo* variants in *KLC1*, one of the candidates from the dystonia trio analysis, are associated with a neurological disease spectrum ranging from movement disorders with comorbid neurodevelopmental signs to more unspecific neurodevelopmental syndromes with intellectual disability and behavioural abnormalities. Combined with published evidence^17^ showing the importance of KLC1ˋs protein domain that contains the patient variants and our modelling and functional studies, the results suggest that perturbations of the interactions between kinesin-1 and its cargo adaptors contribute to the aetiology of this previously unreported disorder.

## Contributors

Conceptualisation and design: M.D., R.A.S., M.Z.; Data analysis and interpretation: E.P., L. O`R., P.H., I.D., M.D., R.A.S., M.Z.; Data collection: E.P., L. O`R., P.H, I.D., M.C., P. Ha., T.B., R.C., C.C., S.F., E.H., R.H.-V.L., E.I., M.J., L.K., H.M., O.M., M.M.-B., N.E.M., A.N., L.O., M.P., A.S., U.S., M.S., M.W., M.K., S.B., J.N., M.S., D.L.G., R.J., M.D., R.A.S., M.Z.; Figures: E.P., L. O`R., P.H., M.D., R.A.S., M.Z.; Writing-original draft: M.D., R.A.S., M.Z.; Writing-review and editing: all authors. Access and verification of all underlying data: E.P., L. O`R., P.H., M.D., R.A.S., M.Z. All authors read and approved the final version of the manuscript.

## Data sharing statement

Genomic sequencing data of dystonia trios are stored in a repository at the Institute of Human Genetics of TUM University Hospital. Anonymised data are available upon reasonable request from the corresponding author Dr. Michael Zech (email: michael.zech@mri.tum.de) according to local ethics review board guidelines. Additional genetic sequencing data presented in this study originate from research and clinical testing in different centres worldwide—requests to access them should be directed to the authors. Crystallographic structure and data have been deposited with the Protein Data Bank with accession code 28IY. Further data that support the biophysical and functional-study findings of this publication, including mutagenesis, scanning fluorimetry, crystallography, fluorescence polarisation measurements, and immunoprecipitation, are available from the authors Prof. Mark Dodding (mark.dodding@bristol.ac.uk) and Prof. Roberto A. Steiner (roberto.steiner@kcl.ac.uk), upon reasonable request.

## Declaration of interests

The authors declare that they have no competing financial and/or non-financial interests related to this study.

## Appendix A Supplementary data

Supplementary materials

Individual 1

Individual 2
