# Supplementary material for: Trio analysis in dystonia identifies de novo KLC1 variants in a kinesinopathy with distinct motor and neurodevelopmental features
Source: eBioMedicine. 2026 Jun 29;129:106358. doi: 10.1016/j.ebiom.2026.106358 (PMC13333304; doi:10.1016/j.ebiom.2026.106358)
Supplement: Supplementary materials [file mmc1.pdf]

SUPPLEMENTARY MATERIAL

Table of contents

Supplementary Methods

Supplementary Figures

Figure S1	Trio-sequencing data analysis workflow and overview of the prioritised constrained genes containing *de novo* candidate variants according to dystonia clinical category
Figure S2	Sequence alignment of mouse and human KLC TPR domains
Figure S3	Three-dimensional structure of the TPR domain of KLC1 and mapping of identified KLC1 missense variants
Figure S4	Brain MRI data for patients with *KLC1 de novo* missense variants at amino acid position Asp253 (individuals 1 and 2)
Figure S5	Raw western blot data for cell-based immunoprecipitation experiments
Figure S6	Schematic depiction of proposed *KLC1* variant-associated pathomechanisms

Supplementary Tables

Table S1	Summary of data collection and refinement statistics for crystallographic analysis
Table S2	Summary of all prioritised *de novo* variants in variation-constrained genes according to the gnomAD version 4 dataset
Table S3	Summary of T_m values at different protein concentrations
Table S4	Summary of 2-way ANOVA of T_m values followed by Dunnett's multiple comparisons test
Table S5	Statistical analysis of JIP1/JIP3 immunoprecipitation experiments based on presented raw data

Supplementary References

Supplementary Methods

Sequencing and variant analysis

Genomic DNA was extracted from peripheral blood leukocytes of all affected individuals and their biological parents. Trio-based sequencing was performed for all individuals; Individual 3 underwent trio whole-genome sequencing (WGS), whereas Individuals 1, 2, 4, 5, and 6 underwent trio whole-exome sequencing (WES). Sequencing was performed at institutional centres or associated laboratories in Munich, Germany; Cincinnati, OH, USA; Geneva, Switzerland; and Rome, Italy. For WES, exome capture was performed using SureSelect Human All Exon v5 or v6 reagents (Agilent Technologies, Santa Clara, CA, USA) or the Twist Bioscience Exome 2.0 kit (Twist Biosciences, South San Francisco, CA, USA), according to established protocols in contributing centres. For WGS, libraries were prepared using the Illumina TruSeq DNA PCR-Free Kit (Illumina, San Diego, CA, USA). Sequencing was performed on Illumina HiSeq 4000 or NovaSeq 6000 platforms, generating 100–150 bp paired-end reads. Sequence reads were aligned to the human reference genome (GRCh37/hg19) using the Burrows-Wheeler Aligner (BWA), and variants were analysed using published algorithms including the Genome Analysis Toolkit (GATK) and SAMtools. Bioinformatic processing was performed using either EVAdb (Exome/Genome Variant Annotation Database; Munich, Germany) or other local in-house GATK best-practice–based pipelines at the respective sequencing centres[1-6]. Prioritised variants were visually verified using the Integrative Genomics Viewer (IGV; Broad Institute, Cambridge, MA, USA). Variant prioritisation was applied across all datasets retaining rare variants absent from or observed at MAF <0.001 in gnomAD v4.1.0 and available in-house sequencing databases, with adequate sequencing support (coverage ≥20x). *De novo* status was confirmed by the absence of the variant in both parental datasets and, where indicated, verified by Sanger sequencing. For Individual 4, in whom a mosaic variant was identified at reduced allele fraction (~23%, 9/39 reads) on trio WES, mosaic *de novo* status was confirmed by Sanger sequencing of the individual's blood-derived DNA. Variant annotation and classification followed standard established procedures, including assessment of population frequency, evolutionary conservation, and *in silico* pathogenicity predictions, with cross-referencing against relevant disease and variant databases.

Immunoprecipitation

HeLa cells (obtained from ATCC, cat. No. ATCC-CCL2) were maintained in DMEM supplemented with 10% FBS, L-glutamine, and penicillin/streptomycin, and cultured in a humidified incubator with 5% CO_2. For immunoprecipitation experiments, 1×10^6 cells were seeded in 10 cm dishes and transfected the following day with plasmids encoding FLAG-JIP1, FLAG-JIP3, and HA-KLC1^{TPR} (wild type or patient-derived variants p.(Asp253Ala), p.(Asp253Gly), p.(Met257Val), p.(Ser389Phe), and p.(Leu470Phe)) using Effectene transfection reagent (Qiagen, cat. No. 301427), according to the manufacturer's instructions. After 24 h, cells were lysed in 1 mL of lysis buffer (25 mM HEPES, pH 7.5; 150 mM NaCl; 0.1% Nonidet P-40; 0.1% Triton X-100; supplemented with protease inhibitor mixture (Merck/Roche, cat. No. 11873580001)) for 10 min, followed by centrifugation at 13,000 × g for 10 min at 4°C. The clarified supernatant was incubated with 30 µL of anti-HA agarose (A2095, Sigma) for 2 h at 4°C on a rotating wheel. Beads were washed four times with lysis buffer, resuspended in 50 µL of buffer, mixed with 15 µL of SDS sample buffer, and boiled. A total of 20 µL of each sample was subjected to SDS–PAGE and analysed by western blotting using anti-FLAG (Merck, cat. No. F1804) and anti-HA (Merck, cat. No. H9658) antibodies. Fluorescent secondary anti-mouse and anti-rabbit antibodies were used for detection, and fluorescence signals were quantified using a LI-COR Odyssey system.

Supplementary Figures

Supplementary Figure 1 Trio-sequencing data analysis workflow and overview of the prioritised constrained genes containing *de novo* candidate variants according to dystonia clinical category

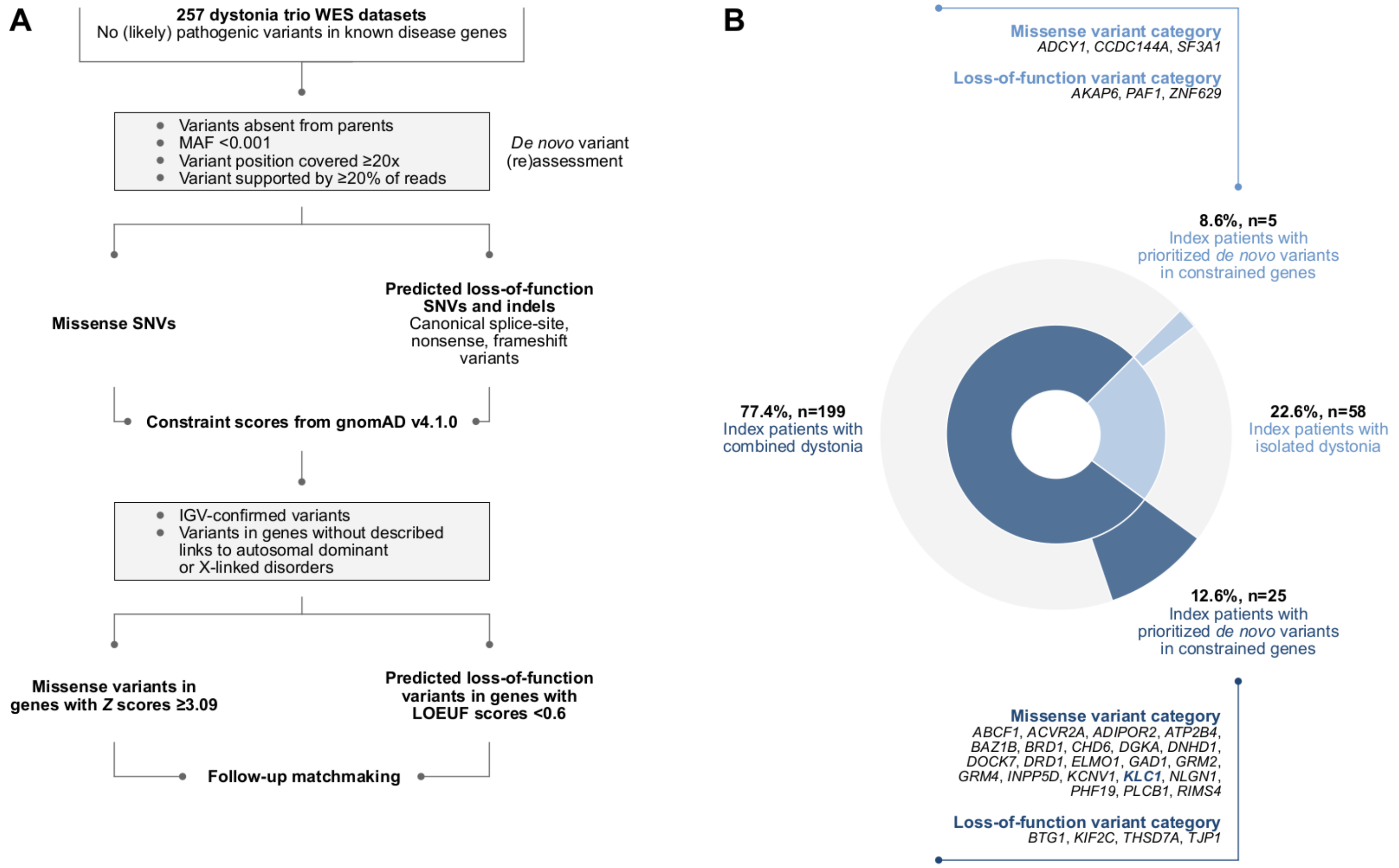

(A) Schematic detailing the evaluation of 257 dystonia trios without likely pathogenic or pathogenic variants in known disease genes[7]. We obtained high-confidence *de novo* variant calls, focusing on rare alterations with a minor allele frequency (MAF) <0.001. Priority was then given to missense single-nucleotide variants (SNVs) in genes with *Z* scores ≥3.09 according to the gnomAD v4.1.0 dataset[8], as well as predicted loss-of-function SNVs and short insertion-deletion variations (indels) in genes with LOEUF (loss-of-function observed/expected upper bound fraction) scores <0.6 (gnomAD v4.1.0)[8]. Only genes with no documented associations to monogenic autosomal dominant/X-linked disorders were considered in this candidate-gene discovery approach. All prioritised *de novo* candidate variants were visually verified using the Integrative Genomics Viewer (IGV), and the affected genes and specific mutational events were subjected to follow-up evaluations implementing different matchmaking strategies[1,2,9]. (B) Breakdown of the two main clinical categories of dystonia[10] and percentages of the patients in whom *de novo* candidate variants were found. The prioritised genes affected by either missense or predicted loss-of-function variants are summarised; the findings are outlined in detail in Suppl.Tab.2.

Supplementary Figure 2 Sequence alignment of mouse and human KLC TPR domains

```
hs_KLC1_TPR      ggyeiparlrtlhnlviqyasqgryevavplckqaledlektsghdhpdvatmlnilalv  265
mm_KLC1_TPR      ggyeiparlrtlhnlviqyasqgryevavplckqaledlektsghdhpdvatmlnilalv  265
                 ************************************************************

hs_KLC1_TPR      yrdqnkykdaanllndalairektlgkdhpavaatlnnlavlygkrgkykeaeplckral  325
mm_KLC1_TPR      yrdqnkykdaanllndalairektlgrdhpavaatlnnlavlygkrgkykeaeplckral  325
                 **************************:*********************************

hs_KLC1_TPR      eirekvlgkdhpdvakqlnnlallcqnqgkyeeveyyyqraleiyqtklgpddpnvaktk  385
mm_KLC1_TPR      eirekvlgkdhpdvakqlnnlallcqnqgkyeeveyyyqralgiyqtklgpddpnvaktk  385
                 ****************************************** *****************

hs_KLC1_TPR      nnlascylkqgkfkqaetlykeiltraherefgsvddenkpiwmhaeereeckgkqkdgt  445
mm_KLC1_TPR      nnlascylkqgkfkqaetlykeiltraherefgsvddenkpiwmhaeereeckgkqkdgs  445
                 ***********************************************************:

hs_KLC1_TPR      sfgeyggwykackvdsptvtttlknlgalyrrqgkfeaaetleeaamrsrk  496
mm_KLC1_TPR      afgeyggwykackvdsptvtttlknlgalyrrqgkfeaaetleeaamrsrk  496
                 :**************************************************
```

Tetratricopeptide repeat (TPR) domain sequence (amino acids 205 to 495) from human (NP_001123579.1) and mouse (NP_032476.2) orthologues of KLC1 were aligned using Clustal Omega[11]. Output is as presented by the software, annotated with arrows to show positions of variants functionally tested in this study.

Supplementary Figure 3 Three-dimensional structure of the TPR domain of KLC1 and mapping of identified KLC1 missense variants

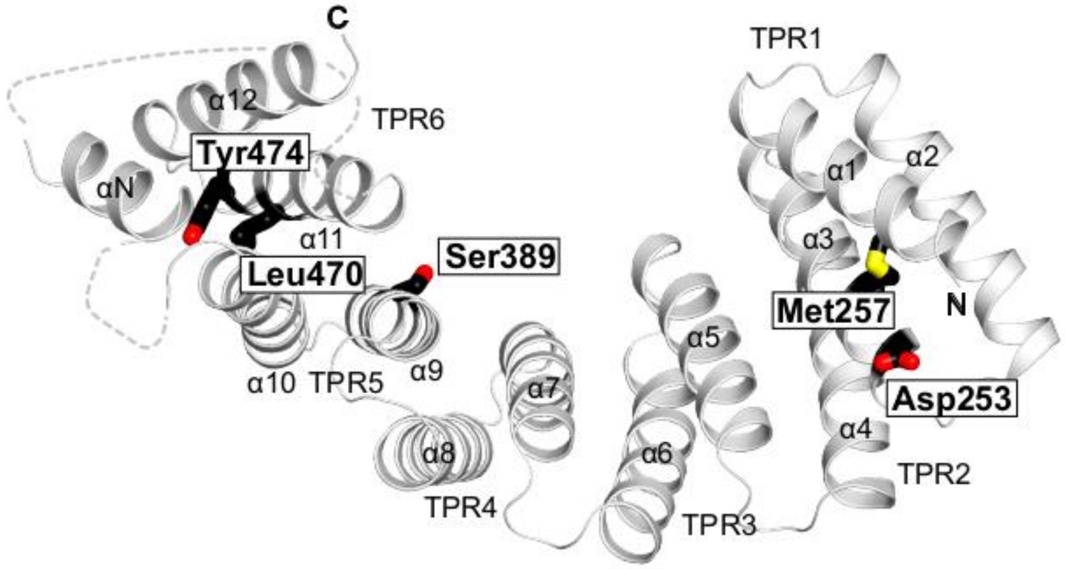

The cartoon representation of the tetratricopeptide repeat (TPR) domain of KCL1 is based on PDB: 3NF1. The domain consists of 6 tetratrico repeats, each composed of 2 antiparallel α-helices connected by a turn with an additional non-TPR helix (αN) located between TPR5 and TPR6. N- and C-termini are labelled with the letters N and C, respectively. Flexible regions are indicated by a dashed line. The 5 amino acids (Asp253, Met257, Ser389, Leu470, Tyr474) involved in the 7 patient-detected variants are shown in black as stick representation.

Supplementary Figure 4 Brain MRI data for patients with *KLC1 de novo* missense variants at amino acid position Asp253 (individuals 1 and 2)

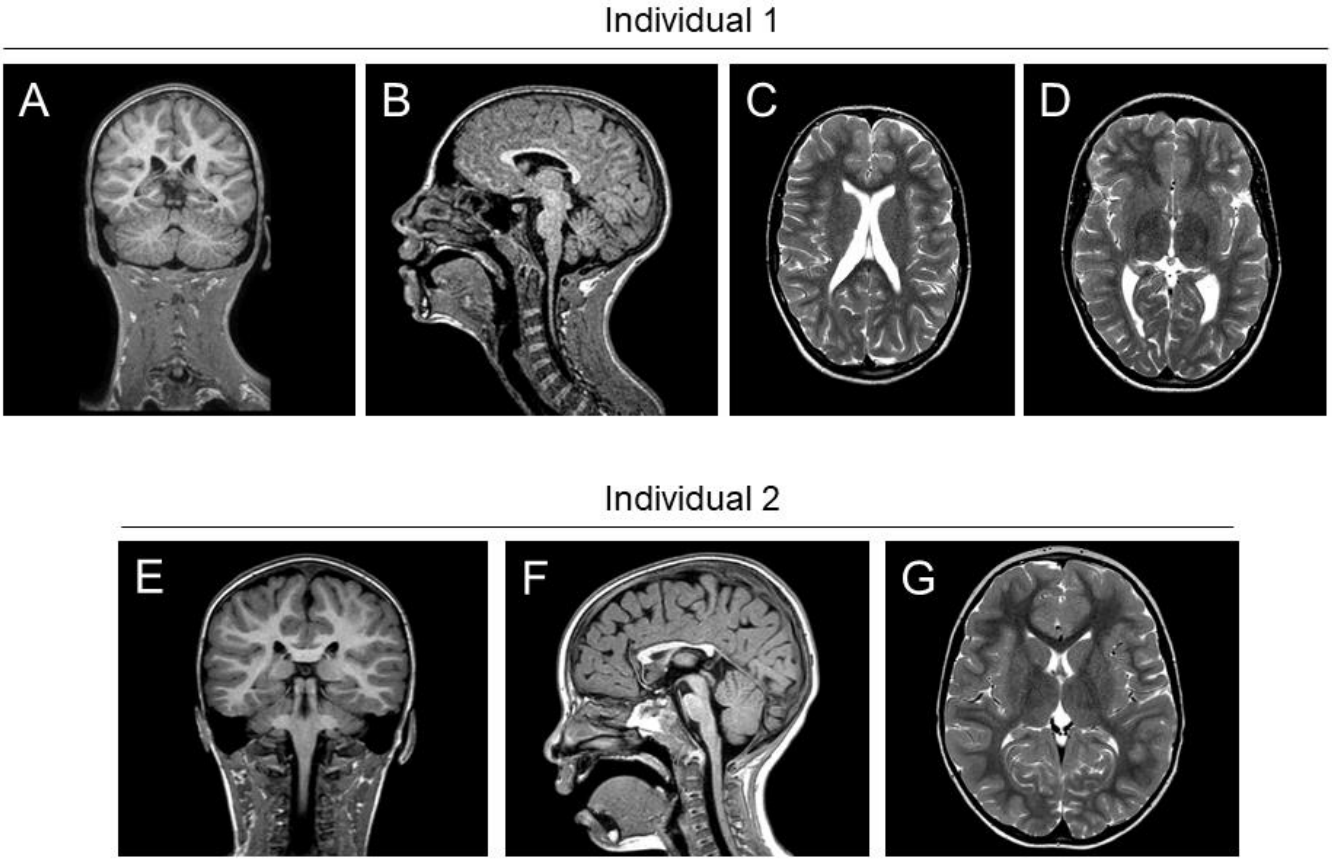

Magnetic resonance imaging (MRI) findings of patients with the recurrently affected amino acid of KLC1 (Asp253) are shown. (A) Coronal T1-weighted, (B) sagittal T1-weighted, and (C, D) axial T2-weighted images of individual 1 with mild cortical atrophy and mild white matter loss. (E) Coronal T1-weighted, (F) sagittal T1-weighted, and (G) axial T2-weighted images of individual 2 with thinning of the corpus callosum and mild cerebral white matter volume loss.

Supplementary Figure 5 Raw western blot data for cell-based immunoprecipitation experiments

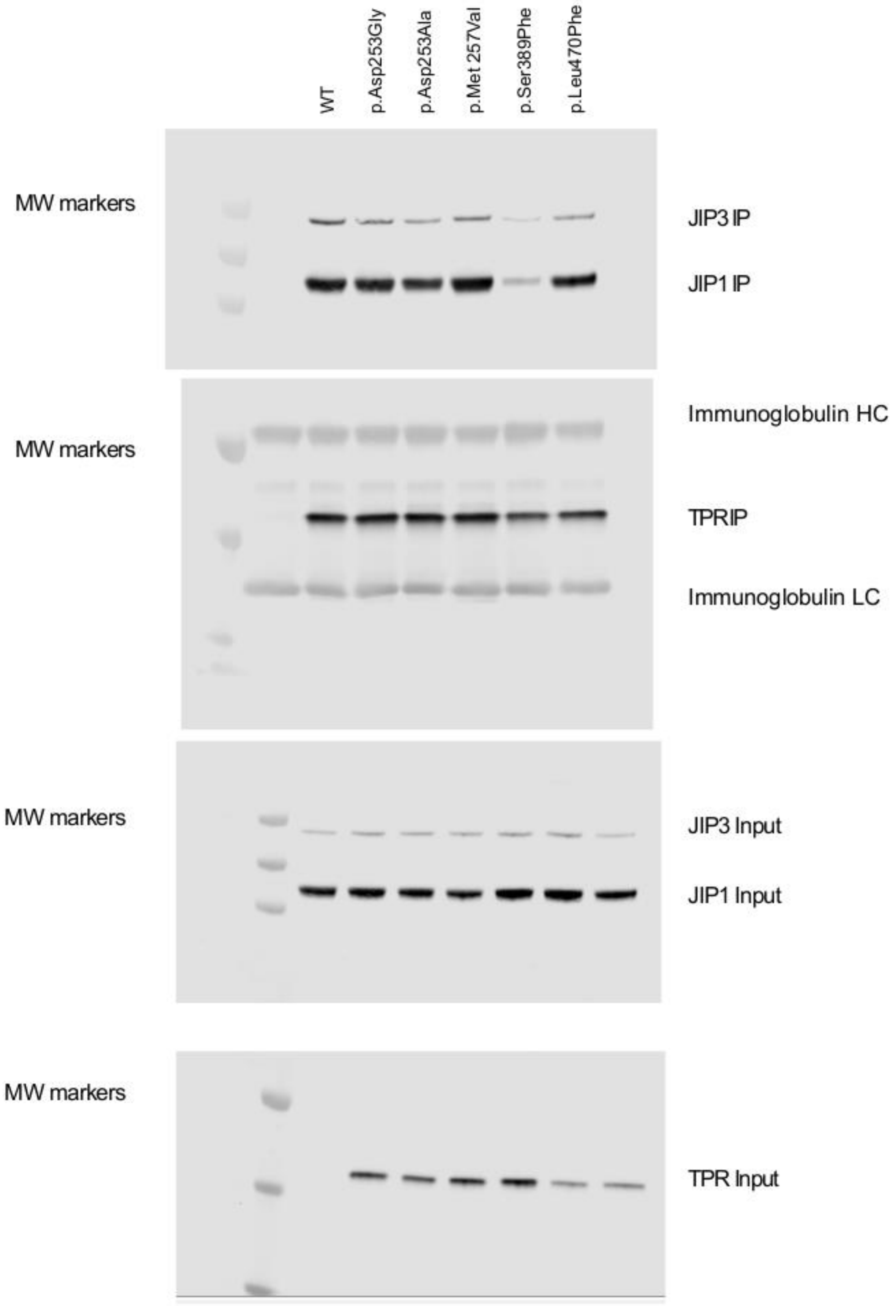

Cell-based assessment of impaired interactions between the tetratricopeptide repeat (TPR) domain of KLC1 and its binding partners JIP1 and JIP3 in the presence of patient-derived KLC1 missense variants. For immunoprecipitation (IP) experiments, HeLa cells were transfected with the indicated HA-TPR, FLAG-JIP1 and FLAG-JIP3 expression constructs, lysed and immunoprecipitated using anti-HA-agarose. Bound proteins (top) and expression in input cell extracts (bottom) were analysed by western blot using anti-HA and anti-FLAG antibodies.

Supplementary Figure 6 Schematic depiction of proposed *KLC1* variant-associated pathomechanisms

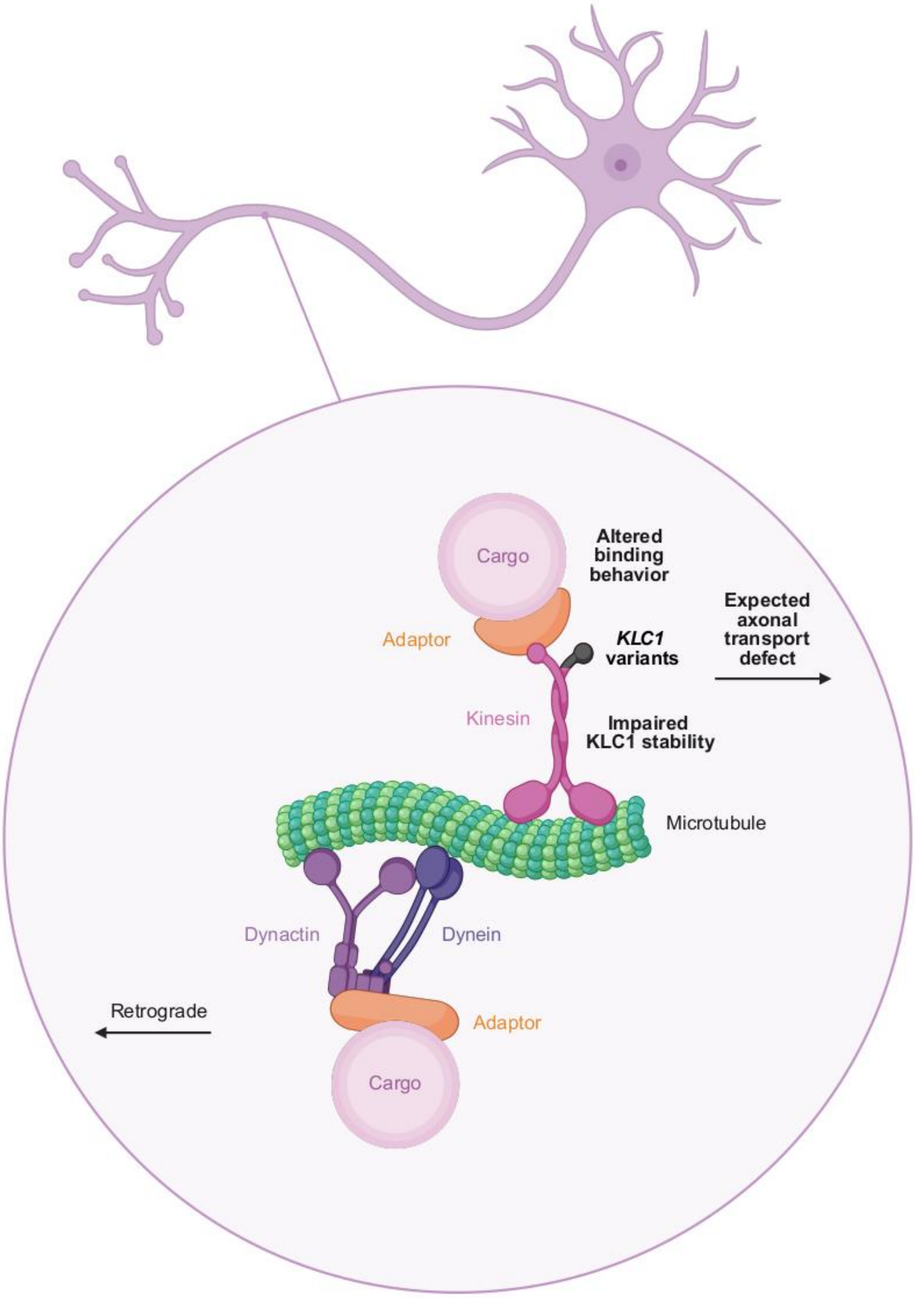

The identified *de novo* missense variants in *KLC1* may impair axonal transport via direct disruption of cargo-adaptor binding to kinesin-1 and/or deleterious KLC1 stability changes.

Supplementary Tables

Supplementary Table 1 Summary of data collection and refinement statistics for crystallographic analysis

Data set	Nb:KLC1^{extTPR}-JIP1$^{C\text{-}term}$
Beam Line	I03 (DLS)
Wavelength (Å)	0.9763
Resolution range[a] (Å)	44.77-2.13 (2.26-2.13)
Space group	*C*2
Cell dimensions (*a*, *b*, *c*) (Å) (α, β, γ) (°)	107.58, 89.54, 51.33 90, 100.56, 90
Unique reflections[a]	21549 (1074)
Overall redundancy[a]	6.5 (7.0)
Completeness ellipsoidal[a] (%)	92.9 (60.5)
Completeness spherical[a] (%)	80.0 (24.7)
R_{merge}[a] (%)	6.2 (132.5)
$R_{p.i.m.}$ (I)[a] (%)	2.6 (53.9)
CC(1/2)	0.996 (0.362)
⟨I/σ(I) ⟩[a]	12.7 (1.3)
**Refinement**	
PDB code	28IY
R_{factor} (%)/R_{free}(%)	22.2/24.4
# non-H atoms	6569
Average *B* value (Å^2)	95.8
rms bond lengths (Å)	0.008
rms bond angles (°)	0.89
Overall MolProbity score[b]	0.78 (100th percentile)

[a]Numbers in parentheses refer to the highest resolution bin.
[b]MolProbity[12] score combines the clashscore, rotamer, and Ramachandran evaluations into a single score, normalised to be on the same scale as X-ray resolution. 100th percentile is the best among structures of comparable resolution; 0th percentile is the worst.

Supplementary Table 2 Summary of all prioritised *de novo* variants in variation-constrained genes according to the gnomAD version 4 dataset

Trio-WES series index patient ID	Sex	Dystonia clinical category	*De novo* variant IGV-confirmed	Gene	Missense *Z* score gnomAD v4.1.0	*De novo* variant (cDNA, protein)	Variant type	Variant frequency gnomAD v4.1.0	Variant CADD score (GRCh37-v1.7)	WES sequencing depth at variant position	Variant allele fraction (%)
**Missense SNVs in genes with Z scores ≥3.09 (gnomAD v4.1.0)**											
Index_Trio_194[1]	male	combined	yes	*ABCF1*	3.38	NM_001025091.2:c.680G>C, NP_001020262.1:p.Gly227Ala	missense	NA	14.48	55	36
Index_Trio_81	female	combined	yes	*ACVR2A*	4.73	NM_001616.5:c.667A>G, NP_001607.1:p.Ile223Val	missense	6/1611644	20.7	61	61
Index_Trio_210	male	isolated	yes	*ADCY1*[3]	4.71	NM_021116.4:c.265G>T, NP_066939.1:p.Gly89Cys	missense	NA	21.7	100	51
Index_Trio_15	male	combined	yes	*ADIPOR2*[3]	3.52	NM_024551.3:c.403T>A, NP_078827.2:p.Cys135Ser	missense	NA	27.2	60	48
Index_Trio_72	female	combined	yes	*ATP2B4*[3]	4.29	NM_001684.5:c.1610G>A, NP_001675.3:p.Cys537Tyr	missense	3/1614188	32.0	132	20
Index_Trio_194[1]	male	combined	yes	*BAZ1B*[3]	4.56	NM_032408.4:c.1823G>T, NP_115784.1:p.Gly608Val	missense	NA	27.2	167	38
Index_Trio_200	female	combined	yes	*BRD1*	3.5	NM_001304808.3:c.3274G>A, NP_001291737.1:p.Gly1092Ser	missense	29/1613486	26.9	54	50
Index_Trio_223[1]	female	isolated	yes	*CCDC144A*	5.03	NM_014695.3:c.4117A>C, NP_055510.1:p.Asn1373His	missense	NA	9.73	63	30
Index_Trio_193	female	combined	yes	*CHD6*[3]	5.54	NM_032221.5:c.964T>A, NP_115597.3:p.Tyr322Asn	missense	NA	27.7	161	44
Index_Trio_196	male	combined	yes	*CHD6*[3]	5.54	NM_032221.5:c.6106G>A, NP_115597.3:p.Asp2036Asn	missense	NA	20.3	51	57
Index_Trio_121	female	combined	yes	*DGKA*	3.55	NM_001345.5:c.1142T>A, NP_001336.2:p.Val381Asp	missense	NA	30.0	78	56
Index_Trio_6	male	combined	yes	*DNHD1*[3]	3.32	NM_144666.2:c.11395C>G, NP_653267.2:p.Leu3799Val	missense	NA	19.93	184	53
Index_Trio_18	female	combined	yes	*DOCK7*	3.88	NM_001367561.1:c.2285T>C, NP_001354490.1:p.Met762Thr	missense	NA	20.2	67	61
Index_Trio_71	male	combined	yes	*DRD1*[3]	3.21	NM_000794.5:c.1030C>G, NP_000785.1:p.Leu344Val	missense	NA	26.0	118	51
Index_Trio_82	male	combined	yes	*ELMO1*	4.09	NM_014800.10:c.1051A>G, NP_055615.8:p.Met351Val	missense	8/1613940	21.0	90	54
Index_Trio_113	male	combined	yes	*GAD1*[3]	3.75	NM_000817.3:c.1218G>C, NP_000808.2:p.Met406Ile	missense	NA	25.9	79	62
Index_Trio_188	male	combined	yes	*GRM2*	3.22	NM_000839.5:c.2219C>T, NP_000830.2:p.Ala740Val	missense	24/1614102	20.5	336	46
Index_Trio_124	male	combined	yes	*GRM4*[3]	3.87	NM_000841.4:c.2548G>A,	missense	1/1614100	32.0	55	55

						NP_000832.1:p.Glu850Lys					
Index_Trio_136	male	combined	yes	*INPP5D*[3]	3.53	NM_001017915.3:c.1924C>G, NP_001017915.1:p.Pro642Ala	missense	NA	28.3	67	40
Index_Trio_146	male	combined	yes	*KCNV1*[3]	4.56	NM_014379.4:c.316A>G, NP_055194.1:p.Ser106Gly	missense	NA	25.6	67	57
**Index_Trio_91**[2]	**female**	**combined**	**yes**	***KLC1***	**3.93**	**NM_001394837.1:c.758A>C, NP_001381766.1:p.Asp253Ala**	**missense**	**NA**	**28.2**	**136**	**37**
Index_Trio_54	male	combined	yes	*NLGN1*	3.39	NM_001365925.2:c.2423T>C, NP_001352854.1:p.Ile808Thr	missense	NA	24.5	99	48
Index_Trio_182	female	combined	yes	*PHF19*	3.43	NM_015651.3:c.929G>A, NP_056466.1:p.Gly310Glu	missense	NA	23.3	79	48
Index_Trio_102	male	combined	yes	*PLCB1*	3.71	NM_015192.4:c.1729G>A, NP_056007.1:p.Glu577Lys	missense	NA	25.9	40	40
Index_Trio_198[1]	male	combined	yes	*RIMS4*[3]	3.42	NM_182970.4:c.106C>T, NP_892015.1:p.Arg36Trp	missense	1/1611680	32.0	152	50
Index_Trio_223[1]	female	isolated	yes	*SF3A1*	4.8	NM_005877.6:c.1219C>G, NP_005868.1:p.Pro407Ala	missense	NA	23.0	118	47

Trio-WES series index patient ID	Sex	Dystonia clinical category	*De novo* variant IGV-confirmed	Gene	LOEUF score gnomAD v4.1.0	*De novo* variant (cDNA, protein)	Variant type	Variant frequency gnomAD v4.1.0	Variant CADD score (GRCh37-v1.7)	WES sequencing depth at variant position	Variant allele fraction (%)
**Predicted loss-of-function SNVs and indels in genes with LOEUF scores <0.6 (gnomAD v4.1.0)**											
Index_Trio_228	male	isolated	yes	*AKAP6*[3]	0.37	NM_004274.5:c.5093_5096del, NP_004265.3:p.Ser1698PhefsTer24	frameshift	NA	NA	134	44
Index_Trio_143	male	combined	yes	*BTG1*[3]	0.46	NM_001731.3:c.403C>T, NP_001722.1:p.Gln135Ter	nonsense	NA	37.0	63	56
Index_Trio_167	female	combined	yes	*KIF2C*[3]	0.43	NM_006845.4:c.1471dup, NP_006836.2:p.Val491GlyfsTer7	frameshift	NA	NA	57	47
Index_Trio_256	female	isolated	yes	*PAF1*	0.596	NM_019088.4:c.1162_1165del, NP_061961.2:p.Glu388LysfsTer169	frameshift	NA	NA	132	45
Index_Trio_50	male	combined	yes	*THSD7A*[3]	0.5	NM_015204.3:c.1981C>T, NP_056019.1:p.Arg661Ter	nonsense	5/1613550	42.0	120	48
Index_Trio_198[1]	male	combined	yes	*TJP1*[3]	0.28	NM_001330239.3:c.1150+1G>T, p.?	splice-site	NA	35.0	126	48
Index_Trio_226	male	isolated	yes	*ZNF629*[3]	0.28	NM_001080417.3:c.1535_1538dup, NP_001073886.1:p.Phe514ProfsTer25	frameshift	NA	NA	431	53

[1]Three index patients from the trio WES series each had 2 prioritised *de novo* variants in 2 different genes under mutational constraint (Index_Trio_194, Index_Trio_198, Index_Trio_223).
[2]Index_Trio_91 corresponds to individual 1 in the present study of *KLC1* variants.
[3]Follow-up case matching and/or functional study efforts underway.

Abbreviations: CADD; Combined Annotation Dependent Depletion; gnomAD; Genome Aggregation Database; IGV, Integrative Genomics Viewer; indel, short insertion and deletion variation; LOEUF, loss-of-function observed/expected upper bound fraction; NA, not available; SNV, single-nucleotide variant, WES, whole-exome sequencing.

Supplementary Table 3 Summary of T_m values at different protein concentrations

	Protein concentration (µM)			
	2	5	10	30
	Mean T_m±SD (°C)			
WT	54.11±0.68	55.62±0.29	54.92±0.17	54.44±0.27
p.Asp253Gly	51.55±0.21	52.10±0.22	51.54±0.16	50.51±0.11
p.Asp253Ala	53.97±1.70	55.11±0.30	54.33±0.17	53.48±0.30
p.Met257Val	57.59±0.07	58.13±0.09	58.13±0.04	56.62±0.10
p.Ser389Phe	53.27±0.15	53.72±0.19	53.32±0.17	52.46±0.34
p.Leu470Phe	50.95±1.49	52.27±0.37	51.50±0.14	49.44±0.31

T_m values were calculated by fitting the melting curves (n=4) to the Boltzmann equation.

Supplementary Table 4 Summary of 2-way ANOVA of T_m values followed by Dunnett's multiple comparisons test

Dunnett's multiple comparisons test	T_m mean difference (°C)	95% CI of difference	Adjusted p-value	Below threshold?	Summary
Protein concentration = 2 μM					
p.Asp253Gly vs WT	-2.560	-3.512 to -1.608	<0.0001	Yes	****
p.Asp253Ala vs WT	-0.140	-1.092 to 0.812	0.9951	No	ns
p.Met257Val vs WT	3.480	2.528 to 4.432	<0.0001	Yes	****
p.Ser389Phe vs WT	-0.840	-1.792 to 0.112	0.1013	No	ns
p.Leu470Phe vs WT	-3.160	-4.112 to -2.208	<0.0001	Yes	****
Protein concentration = 5 μM					
p.Asp253Gly vs WT	-3.520	-4.472 to -2.568	<0.0001	Yes	****
p.Asp253Ala vs WT	-0.510	-1.462 to 0.442	0.5086	No	ns
p.Met257Val vs WT	2.510	1.558 to 3.462	<0.0001	Yes	****
p.Ser389Phe vs WT	-1.900	-2.852 to -0.948	<0.0001	Yes	****
p.Leu470Phe vs WT	-3.350	-4.302 to -2.398	<0.0001	Yes	****
Protein concentration = 10 μM					
p.Asp253Gly vs WT	-3.380	-4.332 to -2.978	<0.0001	Yes	****
p.Asp253Ala vs WT	-0.590	-1.542 to 0.362	0.3696	No	ns
p.Met257Val vs WT	3.210	2.258 to 4.162	<0.0001	Yes	****
p.Ser389Phe vs WT	-1.600	-2.552 to -0.648	<0.0001	Yes	****
p.Leu470Phe vs WT	-3.420	-4.372 to -2.468	<0.0001	Yes	****
Protein concentration = 30 μM					
p.Asp253Gly vs WT	-3.930	-4.882 to -2.978	<0.0001	Yes	****
p.Asp253Ala vs WT	-0.960	-1.912 to -0.008	0.0472	Yes	*
p.Met257Val vs WT	2.180	1.228 to 3.132	<0.0001	Yes	****
p.Ser389Phe vs WT	-1.980	-2.932 to -1.028	<0.0001	Yes	****
p.Leu470Phe vs WT	-5.000	-5.952 to -4.048	<0.0001	Yes	****

Adjusted p-values: * = p<0.05, **** = p<0.0001, ns = not significant.
WT, wild type.

Supplementary Table 5 Statistical analysis of JIP1/JIP3 immunoprecipitation experiments based on presented raw data*

**JIP1 comparison vs wild type**					
Dunnett's multiple comparisons test	Mean diff.	95.00% CI of diff.	Below threshold?	Summary	Adjusted P Value
Wild type vs. p.Asp253Gly	0.2026	-0.1907 to 0.5960	No	ns	0.4617
Wild type vs. p.Asp253Ala	0.05966	-0.3337 to 0.4530	No	ns	0.9886
Wild type vs. p.Met257Val	0.2816	-0.1117 to 0.6750	No	ns	0.2
Wild type vs. p.Ser389Phe	0.9056	0.5123 to 1.299	Yes	****	<0.0001
Wild type vs. p.Leu470Phe	0.07389	-0.3195 to 0.4672	No	ns	0.9719
**JIP3 comparison vs wild type**					
Dunnett's multiple comparisons test	Mean diff.	95.00% CI of diff.	Below threshold?	Summary	Adjusted P Value
Wild type vs. p.Asp253Gly	0.6076	0.2226 to 0.9926	Yes	**	0.0026
Wild type vs. p.Asp253Ala	0.5981	0.2131 to 0.9831	Yes	**	0.003
Wild type vs. p.Met257Val	0.5367	0.1517 to 0.9217	Yes	**	0.0066
Wild type vs. p.Ser389Phe	0.918	0.5330 to 1.303	Yes	****	<0.0001
Wild type vs. p.Leu470Phe	0.1574	-0.2276 to 0.5424	No	ns	0.6558
**Table describing outcomes**					
	Effect on JIP1	Effect on JIP3			
p.Asp253Gly	Not significant	Partial inhibition			
p.Asp253Ala	Not significant	Partial inhibition			
p.Met257Val	Not significant	Partial Inhibition			
p.Ser389Phe	Strong Inhibition	Strong Inhibition			
p.Leu470Phe	Not significant	None			

*Raw data as analysed in GraphPad Prism.

Supplementary References

1. Zech M, Jech R, Boesch S, et al. Monogenic variants in dystonia: an exome-wide sequencing study. *Lancet Neurol* 2020; **19**(11): 908-18.
2. Zech M, Dzinovic I, Skorvanek M, et al. Combined genomics and proteomics unveils elusive variants and vast aetiologic heterogeneity in dystonia. *Brain* 2025; **148**(8): 2827-46.
3. Schmidt A, Danyel M, Grundmann K, et al. Next-generation phenotyping integrated in a national framework for patients with ultrarare disorders improves genetic diagnostics and yields new molecular findings. *Nat Genet* 2024; **56**(8): 1644-53.
4. Neilson DE, Zech M, Hufnagel RB, et al. A Novel Variant of ATP5MC3 Associated with Both Dystonia and Spastic Paraplegia. *Mov Disord* 2022; **37**(2): 375-83.
5. Domenach L, Rooryck C, Legendre M, Bouchghoul H, Beneteau C, Margot H. Antenatal phenotype associated with PAK2 pathogenic variants: bilateral pleural effusion as a warning sign. *BMC Med Genomics* 2025; **18**(1): 35.
6. De Falco A, De Brasi D, Della Monica M, et al. A Novel Variant in RAD21 in Cornelia De Lange Syndrome Type 4: Case Report and Bioinformatic Analysis. *Genes (Basel)* 2023; **14**(1).
7. Hamosh A, Scott AF, Amberger JS, Bocchini CA, McKusick VA. Online Mendelian Inheritance in Man (OMIM), a knowledgebase of human genes and genetic disorders. *Nucleic Acids Res* 2005; **33**(Database issue): D514-7.
8. Chen S, Francioli LC, Goodrich JK, et al. A genomic mutational constraint map using variation in 76,156 human genomes. *Nature* 2024; **625**(7993): 92-100.
9. Sobreira N, Schiettecatte F, Valle D, Hamosh A. GeneMatcher: a matching tool for connecting investigators with an interest in the same gene. *Hum Mutat* 2015; **36**(10): 928-30.
10. Albanese A, Bhatia KP, Fung VSC, et al. Definition and Classification of Dystonia. *Mov Disord* 2025; **40**(7): 1248-59.
11. Sievers F, Wilm A, Dineen D, et al. Fast, scalable generation of high-quality protein multiple sequence alignments using Clustal Omega. *Mol Syst Biol* 2011; **7**: 539.
12. Davis IW, Leaver-Fay A, Chen VB, et al. MolProbity: all-atom contacts and structure validation for proteins and nucleic acids. *Nucleic Acids Res* 2007; **35**(Web Server issue): W375-83.
